# Biogeographic and Evolutionary Patterns of Trace Element Utilization in Marine Microbial World

**DOI:** 10.1016/j.gpb.2021.02.003

**Published:** 2021-02-23

**Authors:** Yinzhen Xu, Jiayu Cao, Liang Jiang, Yan Zhang

**Affiliations:** 1 Shanghai Institute of Nutrition and Health, Shanghai Institutes for Biological Sciences, University of Chinese Academy of Sciences, Chinese Academy of Sciences, Shanghai 200031, China; 2 Shenzhen Key Laboratory of Marine Bioresources and Ecology, College of Life Sciences and Oceanography, Shenzhen University, Shenzhen 518055, China; 3 Shenzhen-Hong Kong Institute of Brain Science-Shenzhen Fundamental Research Institutions, Shenzhen 518055, China

**Keywords:** Trace element, Marine metagenome, Metalloprotein, Selenoprotein, Evolution

## Abstract

**Trace elements** are required by all organisms, which are key components of many enzymes catalyzing important biological reactions. Many trace element-dependent proteins have been characterized; however, little is known about their occurrence in microbial communities in diverse environments, especially the global marine ecosystem. Moreover, the relationships between trace element utilization and different types of environmental stressors are unclear. In this study, we used metagenomic data from the Global Ocean Sampling expedition project to identify the biogeographic distribution of genes encoding trace element-dependent proteins (for copper, molybdenum, cobalt, nickel, and selenium) in a variety of marine and non-marine aquatic samples. More than 56,000 **metalloprotein** and **selenoprotein** genes corresponding to nearly 100 families were predicted, becoming the largest dataset of marine metalloprotein and selenoprotein genes reported to date. In addition, samples with enriched or depleted metalloprotein/selenoprotein genes were identified, suggesting an active or inactive usage of these micronutrients in various sites. Further analysis of interactions among the elements showed significant correlations between some of them, especially those between nickel and selenium/copper. Finally, investigation of the relationships between environmental conditions and metalloprotein/selenoprotein families revealed that many environmental factors might contribute to the **evolution** of different metalloprotein and/or selenoprotein genes in the marine microbial world. Our data provide new insights into the utilization and biological roles of these trace elements in extant marine microbes, and might also be helpful for the understanding of how these organisms have adapted to their local environments.

## Introduction

All living organisms are dependent on various chemical elements. Unlike macroelements (such as hydrogen, carbon, nitrogen, oxygen, and sulfur) which are present in large quantities to build up biological entities, microelements (also known as trace elements) are required only in minute amounts but indispensable for growth, development, and physiology of organisms [1], [2]. The majority of biological trace elements are metals. Among them, iron (Fe) and zinc (Zn) are considered to be used by all organisms [3], [4]. Other metals, such as copper (Cu), manganese (Mn), molybdenum (Mo), nickel (Ni), and cobalt (Co), are thought to act as a vital part of many metalloproteins in a wide range of organisms in the three domains of life [5]. Selenium (Se), the major metalloid micronutrient, also plays an important role in a variety of redox and metabolic processes [6], [7].

The utilization of trace elements in different organisms is very complex. Most metals are directly used as cofactors inserted into their cognate sites in proteins, whereas Mo and Co are mainly present in the forms of molybdopterin (Mo cofactor, Moco) and vitamin B_12_ (cobalamin), respectively [8], [9]. The number of metalloprotein families also varies greatly depending on which metal is used [10], [11], [12]. The utilization of Se is different from other trace elements, which mainly exists as selenocysteine (Sec, a non-standard amino acid encoded by UGA codon) and is found in all selenoproteins [13].

In recent years, a rapid increase in the number of genome sequencing projects (especially microorganisms) has led to the production of a huge amount of genomic data. Most biological processes and proteins involved in trace element metabolism and function (*e.g.*, transporters, cofactor biosynthesis components, and trace element-dependent proteins) have been either first or best characterized in various prokaryotes. Previous studies have shown that bacteria have a much more active usage of trace elements than eukaryotes [14], [15], [16], [17], [18], [19], [20]. Preliminary analysis of the relationship between environmental conditions and trace element-dependent proteins revealed that different habitats may affect not only the distribution of individual proteins but also the complete sets of metalloproteins (metalloproteome) and selenoproteins (selenoproteome). It has been reported that certain environmental factors (such as dissolved oxygen levels and water temperature) may generally correlate with large size of metalloproteomes (such as Mo, Co, and Cu) or selenoproteomes in many aquatic microbes [14], [15], [16], [17], [18], [19]. However, the question whether different types of aquatic environments can influence either the use of individual trace elements or their interactions is largely unexplored.

The Earth’s oceans contain a great number of microorganisms which remain elusive because only a small part of microbes can be cultivated and studied in the laboratory. Nowadays, metagenomic analysis has become more and more important for understanding the species composition and diversity in a natural sample including marine ecosystem. Several studies have investigated the relation between trace element utilization and various oceanic environments, which implies that marine biogeochemical cycles and trace metal utilization have co-evolved and could influence each other [21], [22]. It has been suggested that gene loss, metal substitution, and lateral gene transfer have been important in shaping metal utilization of extant marine microbes [23], [24]. For example, using part of the metagenomic data from the Global Ocean Sampling (GOS) expedition which is the largest marine metagenomic study performed over a geographically wide sea area [25], several studies analyzed selenoprotein genes and Fe uptake genes in surface ocean microbes [26], [27], [28], [29], which provided a first glance at the metabolism and roles of these micronutrients in marine microbial communities. However, due to limited sequence resources available at that time, the majority of the GOS datasets derived from much more selected locations have not been examined yet.

In this study, we reported a comparative metagenomic analysis of five biological trace elements (including Cu, Mo, Ni, Co, and Se) in marine microbial communities by using the most updated GOS shotgun sequence dataset from hundreds of diverse aquatic (largely marine) sites. The biogeographic distribution and abundance of all known metalloprotein genes related to these metals and selenoprotein genes were analyzed. More importantly, we assessed the effects of a variety of aquatic environmental factors (including natural environmental features and human impacts) on the utilization and function of these elements. Our data offer new insights into evolutionary trends of trace element utilization in the marine microbial world.

## Results

A diagram of the workflow is shown in Figure S1. Using the GOS metagenomic data combined with environmental information (details are shown in Table S1), we generated a large map which illustrates the patterns of trace element utilization in a global biogeographical context. More than 56,000 metalloprotein and selenoprotein genes were predicted, which is so far the largest dataset of genes encoding trace element-dependent proteins. All predicted metalloprotein and selenoprotein sequences are listed in File S1.

### Distribution of metalloprotein genes and metalloproteomes

**Figure 1:**
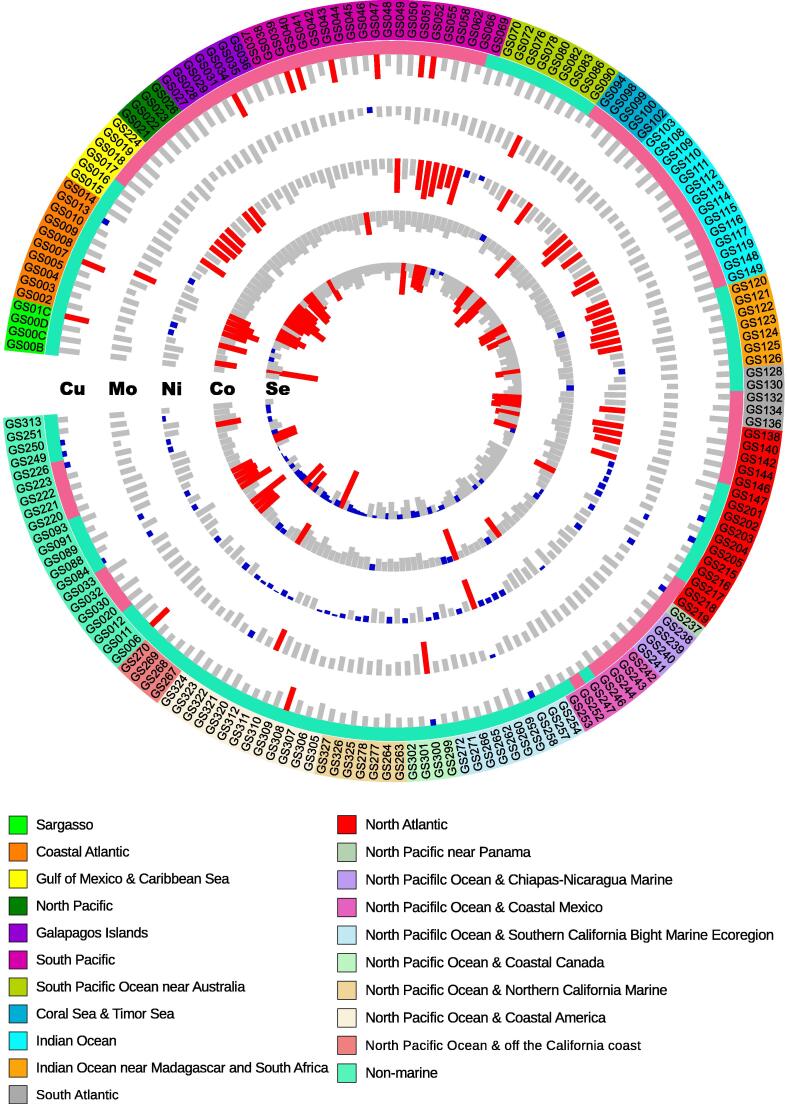
**General distribution of metalloproteomes and selenoproteomes in GOS samples** The five tracks (circles) within the GOS sample ID circle (from outside to inside) represent the normalized occurrence of metalloproteomes (Cu, Mo, Ni, and Co) and selenoproteomes, respectively, based on the geographical location across the global oceans. The length of each column represents the normalized ratio of the fraction of metalloproteome/selenoproteome in each sample to the average of corresponding proteomes. Metalloprotein/selenoprotein-rich and -poor samples are highlighted in red and blue, respectively. Sequential color schemes represent different geographical groups (totally 21 groups). Tropical and temperate regions are indicated in pink and light green, respectively. GOS, Global Ocean Sampling.

Homology-based analysis of 179 GOS samples revealed a large number of metalloprotein genes for Cu, Mo, Ni, and Co in marine microbial communities (Table 1). These samples were initially clustered in straightforward ways such as geographical location, temperature, and salinity. The general distribution of GOS metalloproteomes is shown in Figure 1.

**Table 1:** **Distribution of metalloprotein and selenoprotein genes in the GOS dataset**

**Element**	**No. of genes**	**No. of samples rich in trace element-dependent proteins**	**No. of samples poor in trace element-dependent proteins**
Cu	9985	11	10
Mo	29,219	4	7
Ni	3051	34	42
Co	9592	20	11
Se	4324	36	41

*Note*: GOS, Global Ocean Sampling.

#### Cu

Cu functions as an important catalytic cofactor for several key enzymes. Here, we identified 9985 genes that belong to all known cuproprotein gene families. The fraction of cuproproteomes in individual samples is shown in Figure S2 (details are shown in Table S2). Cytochrome c oxidase subunit I (COX I), COX II, plastocyanin, and Cu-Zn superoxide dismutase (Cu-Zn SOD) were the most commonly used cuproprotein families, whose coding genes were detected in all or almost all examined GOS samples and accounted for 93.1% of all cuproprotein gene sequences. In contrast, less than 50 genes were observed for nitrite reductase (NiR), tyrosinase, particulate methane monooxygenase (pMMO), nitrous oxide reductase, and nitrosocyanin in the whole GOS dataset, implying that these cuproproteins are rarely used by marine microbes.

By identifying the cuproproteome in each sample, 11 cuproprotein-rich and 10 cuproprotein-poor samples were identified (Figure S2; Table S2). The majority of cuproprotein-rich samples (7 out of 11) were collected from the tropical South Pacific Ocean (including Galapagos Islands marine ecoregion). On the contrary, most cuproprotein-poor samples are geographically distant and were collected from either non-marine or temperate regions. Our results suggest that increased water temperature may promote Cu utilization in marine microorganisms while non-marine and/or colder aquatic environments could restrict the utilization of this metal.

#### Mo

Molybdoproteins play central roles in many biological processes of carbon, nitrogen, and sulfur metabolism, including sulfite oxidase (SO), xanthine oxidase (XO), dimethylsulfoxide reductase (DMSOR), and Fe–Mo-containing nitrogenase. Members of molybdoprotein families have been characterized in many organisms [14], [30], [31]. In this study, a total of 29,219 molybdoprotein genes were identified (Table S3), and the distribution of molybdoproteomes in GOS samples is shown in Figure S3. Genes encoding members of SO, XO, and DMSOR families could be detected in all or nearly all GOS samples. XO and DMSOR were the most abundant molybdoprotein families (42.1% and 39.5% of all molybdoprotein sequences, respectively), which is generally consistent with previous observations that organisms containing DMSOR and XO favor aerobic and aquatic conditions [14]. In contrast, only seven nitrogenase genes were found, suggesting that this enzyme is not essential for almost all aerobic marine bacteria.

**Figure 2:**
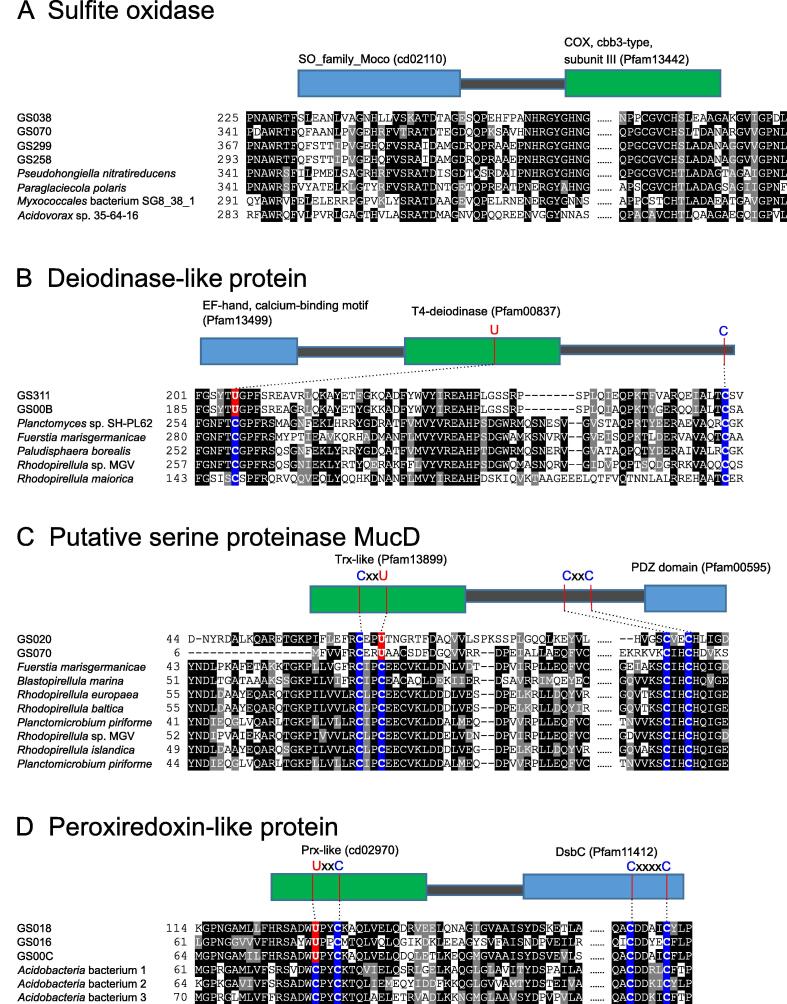
**New domain fusions involving molybdoproteins and selenoproteins** **A.** Sulfite oxidase fused with a cbb3-type COX subunit III domain. **B.** Deiodinase-like protein fused with the EF-hand calcium-binding domain. **C.** Putative serine proteinase MucD fused with a PDZ domain. **D.** Peroxiredoxin-like protein fused with a disulphide bond isomerase domain DsbC. COX, cytochrome c oxidase.

A new domain fusion form of SO was identified in several GOS samples, in which SO is fused with a cbb3-type COX subunit III domain (Pfam13442). Such a fusion form was also present in a small number of aquatic bacterial genomes (Figure 2A). It has been previously reported that the aa3-type COX might catalyze sulfite oxidation in some bacteria [32]. Here, our finding suggests that the cbb3-type COX might also be involved in sulfite oxidation in certain microbes.

Variations in the size of molybdoproteomes were relatively small across GOS samples. We only identified four molybdoprotein-rich samples: GS008 (Coastal Atlantic, close to Newport Harbor, USA), GS080 (under Harbor Bridge, Sydney, Australia), GS300 (North Pacific Ocean & Coastal Canada), and GS311 (North Pacific Ocean & Coastal America). On the other hand, seven samples including GS046 (South Pacific Ocean), GS084 (Lake Tyrrell, Australia), GS089 (Cheetam Salt ponds, Australia), GS205 (North Atlantic Ocean, Gulf of Maine, USA), GS217 (North Atlantic Ocean), GS260 (North Pacific Ocean & Southern California Bight Marine Ecoregion), and GS321 (North Pacific Ocean & Puget Sound, Gedney Island, USA) were molybdoprotein-poor samples. All molybdoprotein-rich and most molybdoprotein-poor samples are derived from temperate marine environments, implying that temperature and salinity are not important factors affecting the size of molybdoproteomes.

#### Ni

Ni is mainly present in a limited number of enzymes that catalyze key reactions in energy and nitrogen metabolism [33]. Here, a total of 3051 genes encoding Ni-dependent proteins were detected (Table S4), and the distribution of Ni-dependent metalloproteomes is shown in Figure S4. Urease and Ni-containing superoxide dismutase (NiSOD) were the most frequently used Ni-dependent proteins, whose coding genes accounted for 54.2% and 34.8% of all Ni-dependent protein genes, respectively. The occurrence of genes responsible for other Ni-dependent enzymes was quite limited. No methyl-coenzyme M reductase sequence could be found in the whole GOS dataset, which is consistent with the idea that this Ni-binding protein is specific for methanogenic archaea [18].

Compared with Cu and Mo, a highly variable distribution of Ni-dependent metalloproteomes was observed in the GOS dataset: 34 Ni-dependent protein-rich and 42 Ni-dependent protein-poor samples were found. About 73.5% of Ni-dependent protein-rich samples are located in the tropical zone, whereas 90.5% of Ni-dependent protein-poor samples belong to the temperate zone, suggesting that increased temperature may stimulate the evolution of Ni-dependent protein-coding genes. We also noticed that almost all Ni-dependent protein-poor samples are close to the continent; however, the majority of Ni-dependent protein-rich samples are located in the open ocean, suggesting that the distance between the sampling site and the continent might be another factor shaping the utilization of this transition metal.

#### Co

Co mainly serves as the metal center of coenzyme B_12_, a complex organometallic cofactor which is present in a variety of enzymes, such as methylmalonyl-CoA mutase (MCM), B_12_-dependent class II ribonucleotide reductase (RNR II), methionine synthase (MetH), B_12_-dependent methyltransferases, and some newly characterized enzymes [9], [34], [35], [36], [37], [38]. Here, we identified 9592 Co-dependent protein genes in the GOS dataset (Table S5), and the distribution of Co-dependent metalloproteomes is shown in Figure S5. Surprisingly, the majority (61.7%) of these sequences belong to RNR II, whose number is much larger than those of the second (MCM, 18.2%) and the third (MetH, 10.6%) most common Co-dependent protein families. Although a previous study has shown that MetH, RNR II, and MCM are the most abundant Co-dependent proteins in sequenced bacteria [17], such a big difference observed in this study needs to be further investigated. Analysis of possible taxonomic affiliation for RNR II genes revealed that 41.1% of them originated from viruses (Figure S6). It has been reported that the GOS dataset contains a relatively high abundance of viral sequences [39]. The prevalence of genes encoding RNR II proteins among viral sequences implies that viral-mediated, Co-dependent nucleotide biosynthesis is an important mechanism for generating microbial diversity in the marine environment.

We identified 20 Co-dependent protein-rich and 11 Co-dependent protein-poor samples. Nearly half of the Co-dependent protein-rich samples are non-marine samples, including 7 saline and hypersaline samples (GS011 and GS012: estuaries of Virginian Marine Ecoregion in North Atlantic Ocean; GS033: Punta Cormorant lagoon in Galapagos; GS084: Lake Tyrell in Australia; GS088 and GS089: Cheetam Salt ponds in Australia; GS249: Isla Carmen in Mexico) and a freshwater sample from Lake Gatun in Panama (GS020). Although a significant overlap of Ni and Co utilization traits in prokaryotes has been previously reported [17], the majority of both Co-dependent protein-rich and Co-dependent protein-poor samples are located in various places of the temperate zone, suggesting a different effect of ocean temperature on Co-dependent metalloproteomes in marine microbes when compared with Ni.

### Distribution of selenoprotein genes and selenoproteomes

Previous analyses of Se utilization in both sequenced organisms and environmental samples have revealed that aquatic habitat (*e.g.*, marine environments) may promote the evolution of new selenoprotein genes [19], [26]. Here, with a significantly increased number of samples, we reanalyzed the occurrence and composition of selenoproteomes in the current GOS dataset. The general distribution of GOS selenoproteomes is shown in Table 1 and Figure 1.

Computational analysis of all samples identified 4324 selenoprotein genes belonging to 59 previously described selenoprotein gene families (Table S6), and the fraction of selenoproteomes in different samples is illustrated in Figure S7. Genes encoding the top 20 selenoprotein families accounted for more than 90.3% of all selenoprotein genes. The prominent selenoproteins include selenoprotein W (SelW)-like protein (11.3%), AhpD-like protein (9.5%), selenophosphate synthetase (SelD, 9.1%), UGSC-containing protein (8.2%), peroxiredoxin (Prx, 7.1%), proline reductase (6.4%), and a variety of Prx- and thioredoxin (Trx)-like proteins.

Previous analysis of part of the GOS dataset revealed multiple domain fusion events involving selenoproteins, which highlights redox activities and key cysteine (Cys) residues of these proteins [26]. Here, we identified new fusion forms of several other selenoproteins (Figure 2B–D), including 1) deiodinase-like protein fused with the EF-hand calcium-binding domain (Pfam13499); 2) putative serine proteinase MucD (containing CxxC/U motif) fused with a PDZ domain (Pfam00595); and 3) Prx-like protein (containing U/CxxC motif) fused with a disulfide bond isomerase domain DsbC (Pfam11412). Interestingly, additional conserved Cys residues without clear function were detected in each of them, indicating that some of them may have a thiol-based redox function.

A total of 36 selenoprotein-rich and 41 selenoprotein-poor GOS samples were identified, which were dispersedly distributed in different oceanic regions (Figure S7). Consistent with previous observations [26], 83.3% of samples from the Gulf of Mexico and the Caribbean Sea (GS015–GS019) showed increased levels of selenoprotein genes. In contrast, 61.9% of the non-marine samples were selenoprotein-poor samples, including GS088 and GS249, in which no known selenoprotein gene could be detected. Approximately 63.9% of selenoprotein-rich samples are located in the tropical zone, while 85.4% of selenoprotein-poor samples are located in the temperate zone, which is consistent with the previous hypothesis that increased temperature might preserve or even stimulate the use of Sec [26], [40].

### Interactions among trace element utilization

Although the utilization of different trace elements appears to be quite distinct from each other, integrated analysis of metalloprotein and selenoprotein genes across all samples or samples with enriched or depleted metalloprotein/selenoprotein genes (*i.e.*, “-rich” or “-poor” samples) may help to better understand the interactions among different trace element utilization in marine microbial communities.

**Figure 3:**
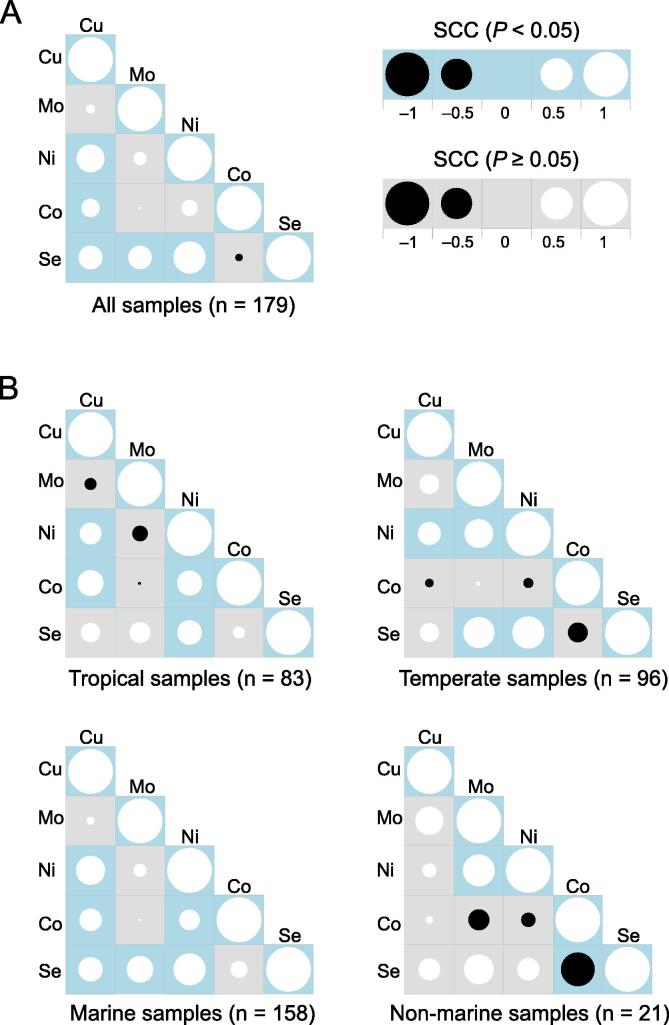
**Correlation analysis of trace element utilization** **A.** All samples. **B.** The tropical, temperate, marine, and non-marine subgroups of samples. Positive and negative correlations are represented in white and black, respectively. The size of the circle is proportional to the SCC values. SCC, Spearman correlation coefficient.

Based on the fractions of metalloprotein and selenoprotein genes in each sample, Spearman correlation coefficient (SCC) was calculated to evaluate the relationship between different trace elements. Five element pairs (Cu–Ni, Cu–Co, Cu–Se, Mo–Se, and Ni–Se) were found to be significantly and positively correlated (Figure 3A; *P* < 0.05). We further divided all samples into several categories: tropical *vs* temperate and marine *vs* non-marine subgroups (Figure 3B). First, element pairs present in Figure 3A were also positively correlated in different subgroups, especially Cu–Ni and Ni–Se, which were present in all subgroups except the non-marine samples. Second, additional significantly correlated element pairs were found in one or more subgroups, such as Co–Ni (in the tropical and marine subgroups) and Ni–Mo (in the temperate and non-marine subgroups). In contrast, a negative correlation was only observed between Co and Se in the non-marine subgroup, implying a contradictory trend in the utilization of these two elements in non-marine aquatic samples.

**Figure 4:**
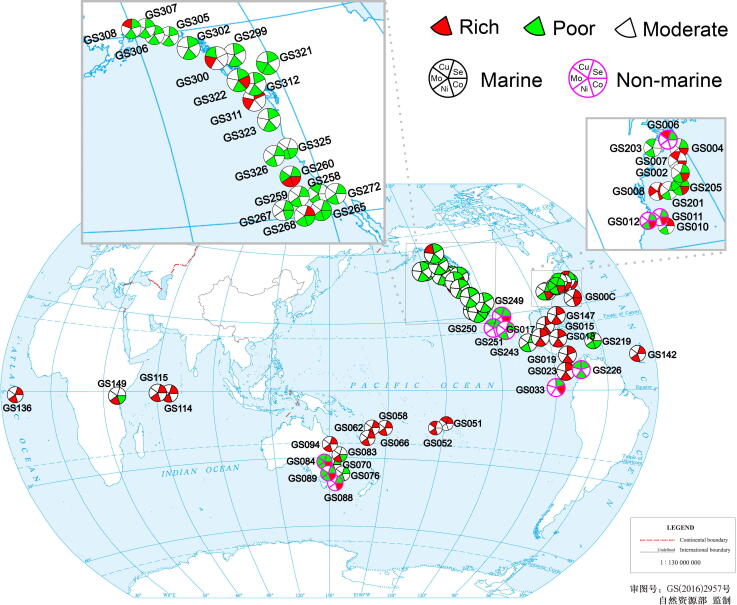
**Geographic locations of GOS samples** All examined samples are shown on the map. Only samples that are trace element-dependent protein-rich/-poor samples for at least two of the five elements are highlighted with pie graphs. Each sample is plotted based on its latitude and longitude coordinates. The base world map is from China Standard Map Service (http://bzdt.ch.mnr.gov.cn/) and has not been modified.

We then focused on “-rich” and “-poor” samples representing either highly active or restricted utilization of certain element (132 samples in total). Among them, 63 samples were trace element-dependent protein-rich/-poor samples involving multiple elements. A geographic distribution map of these samples is shown in Figure 4. The majority of them showed consistent trends for different element utilization (*i.e.*, 20 and 24 samples for metalloprotein/selenoprotein-rich and -poor samples, respectively) (Table 2). For example, 20 samples mainly derived from the Coastal Atlantic (North America), Gulf of Mexico, Galapagos Islands, South Pacific Ocean, Indian Ocean, and North Atlantic Ocean (GS00C, GS007, GS008, GS015, GS017–GS019, GS023, GS051, GS052, GS058, GS062, GS066, GS094, GS114, GS115, GS136, GS142, GS147, and GS311) showed the most active trace element utilization among all examined samples. GS008 was the only marine sample that has a highly active utilization of three elements: Mo, Co, and Se. In contrast, GS219 (a reef sample derived from the Eastern Caribbean close to US Virgin Islands), GS226 (a fresh-water sample from Lake Gatun, Panama), GS265 (a coastal sample from the coast between Tijuana, Mexico and San Diego, CA, USA), and GS321 (a coastal sample from Puget Sound, Gedney Island, USA) appeared to have quite limited utilization of three different elements. Interestingly, 25 out of 31 Ni-dependent protein-poor samples shown in Table 2 were selenoprotein-poor samples, while 14 out of 18 Ni-dependent protein-rich samples were selenoprotein-rich samples, implying that certain factors could activate or inhibit the use of both elements in these areas.

**Table 2:** **Distribution of trace element-dependent protein-rich/-poor samples involving two or more trace elements**

**Sample ID**	**Cu**	**Mo**	**Ni**	**Co**	**Se**	**Sample ID**	**Cu**	**Mo**	**Ni**	**Co**	**Se**
GS00C				+	+	GS149			+	−	
GS002			−	+	−	GS201			−		−
GS004				+	−	GS203	−		−		
GS006	+				−	GS205	−	−	−	+	−
GS007	+			+		GS219	−		−	−	
GS008		+		+	+	GS226	−		−		−
GS010			−		+	GS243			−		−
GS011				+	−	GS249	−			+	−
GS012			−	+	−	GS250	−				−
GS015			+		+	GS251			−		−
GS017			+		+	GS258	−		−		
GS018			+		+	GS259			−		−
GS019			+		+	GS260		−	+	+	−
GS023			+		+	GS265			−	−	−
GS033				+	−	GS267				−	−
GS051	+				+	GS268			−	−	+
GS052	+		+			GS272			−		−
GS058			+		+	GS299			−		−
GS062			+		+	GS300	−	+			
GS066			+		+	GS302			−		−
GS070			−		−	GS305			−		−
GS076			−		−	GS306			−		−
GS083			+	−		GS307			−		−
GS084	−	−	−	+	−	GS308	+		−		−
GS088				+	−	GS311		+			+
GS089		−	−	+	−	GS312			−		−
GS094			+		+	GS321		−	−		−
GS114			+		+	GS322			−	+	−
GS115			+		+	GS323			−		−
GS136			+		+	GS325			−	−	
GS142			+		+	GS326			−		−
GS147			+		+						

*Note*: ‘+’ and ‘−’ represent trace element-dependent protein-rich and -poor samples, respectively.

Some other GOS samples (19 samples) had opposite trends in the use of different trace elements (Table 2). Both GS084 and GS205 were found to have an active utilization of Co; however, utilization of the other four elements was restricted. This may imply a negative correlation between Co-dependent metalloproteins and proteins dependent on other elements in those samples. Similarly, GS089 was also found to be enriched in Co-dependent metalloproteins but had restricted utilization of Mo, Ni, and Se. These results suggest that the biological utilization of trace elements is quite complex and might have been affected by various conditions.

### Correlation between marine environmental factors and trace element utilization

Previous analyses of GOS and some other marine environmental samples have revealed a complex relationship between gene functional compositions and different environmental factors [27], [41], [42], [43], [44]. It is clear that environmentally induced alteration of microbial community structure and diversity may have a direct effect on the metabolism of these organisms, such as trace element utilization, which may be mainly reflected by a significant change in the use of trace element-dependent proteins. Our results described above have also suggested that certain environmental features (such as temperature) may influence the utilization of multiple elements as well as their interactions.

To systematically evaluate the effect of different environmental variables across samples, we first adopted a strategy that was used by Patel et al. [42] to study the sample–sample correlation (SSC) on the basis of environmental variables (SSC-env; see Materials and methods). We constructed an environmental feature matrix in which each GOS sample was represented by a vector of 18 environmental features. SSC-env calculation and sample clustering analysis suggested a distinct latitudinal influence on these samples (Figure S8). This is consistent with the previous observation that seawater temperature (one of the most significant factors related to latitude) might be one of the most important factors across GOS sites [42].

To further investigate the relationship between latitudinal features and metalloprotein/selenoprotein families, we compared the occurrence of genes encoding each of those families in different groups of samples using the Wilcoxon rank-sum test (families that were detected in less than 10 samples were excluded). Several metalloprotein and selenoprotein families were found to be differentially distributed between tropical and temperate samples (Table 3; *P* < 0.05). The majority of them had increased gene levels in tropical-zone samples, especially for Ni and Se. The most abundant Ni-dependent proteins and selenoproteins favored tropical conditions, which may partially explain the strong correlation observed between latitude and their utilization. The occurrence of genes encoding six cuproprotein families was significantly different between tropical and temperate regions, including three (COX I, COX II, and plastocyanin) enriched in tropical samples and the other three (NiR, Cu-Zn SOD, and pMMO) in temperate samples. Thus, the relationship between Cu utilization and temperature observed above is likely due to the fact that the top three most abundant cuproprotein families favor tropical environments. On the other hand, although the distribution of SO and DMSOR appeared to be related to latitude, their fold changes were slight, suggesting that Mo utilization and the evolution of molybdoprotein genes might not be significantly affected by latitudinal features.

**Table 3:** **Differentially distributed metalloprotein and selenoprotein families between tropical and temperate samples**

**Trace element**	**Protein family**	**Fold change** **(tropical *vs* temperate)**
Cu	Cytochrome c oxidase subunit I	1.20
	Cytochrome c oxidase subunit II	1.27
	Nitrite reductase	0.28
	Plastocyanin	1.95
	Cu-Zn superoxide dismutase	0.60
	Particulate methane monooxygenase	0.10

Mo	Sulfite oxidase	0.87
	Dimethylsulfoxide reductase	0.85

Ni	Urease	1.60
	Ni-containing superoxide dismutase	2.02

Co	LitR/CarH/CarA protein	0.37
	Methionine synthase	1.57
	PpaA protein	2.39
	Epoxyqueuosine reductase	0.32

Se	Alkylhydroperoxidase-like	1.67
	5ʹ-nucleotidase/2ʹ,3ʹ-cyclic phosphodiesterase	1.55
	Hypothetical protein GOS_B	2.74
	Peroxiredoxin	1.53
	Selenophosphate synthetase	1.59
	Selenoprotein W-like protein	1.77
	Thioredoxin-like protein	1.84
	UGSC-containing protein	1.18
	Distant homolog of thioredoxin-like protein	2.51

**Figure 5:**
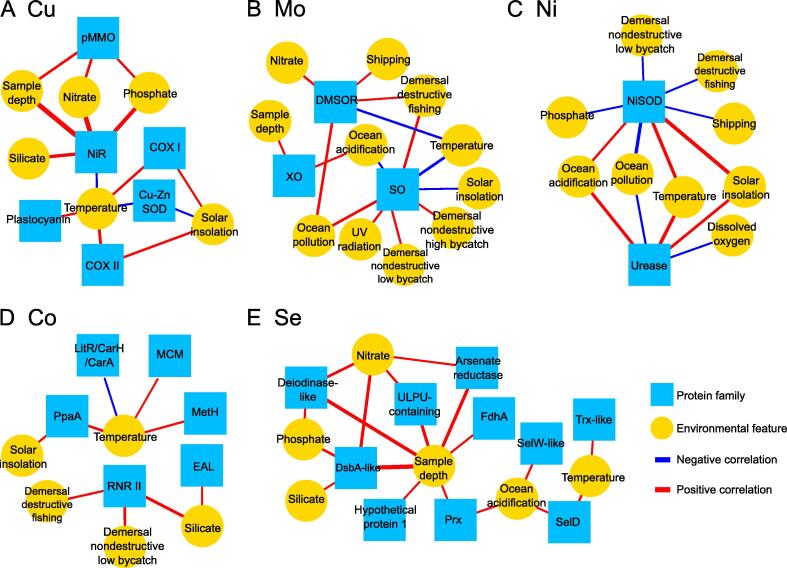
**The relevance network with significant correlations between environmental factors and metalloprotein/selenoprotein families** **A.** Cu. **B.** Mo. **C.** Ni. **D.** Co. **E.** Se. Only significant correlations are showed. The thickness of edges represents the magnitude of association scores. pMMO, particulate methane monooxygenase; NiR, nitrite reductase; COX I, cytochrome c oxidase subunit I; COX II, cytochrome c oxidase subunit II; Cu-Zn SOD, Cu-Zn superoxide dismutase; SO, sulfite oxidase; XO, xanthine oxidase; DMSOR, dimethylsulfoxide reductase; NiSOD, Ni-containing superoxide dismutase; MCM, methylmalonyl-CoA mutase; RNR II, B_12_-dependent class II ribonucleotide reductase; MetH, methionine synthase; EAL, ethanolamine ammonia lyase; DsbA-like, disulfide bond forming protein A-like; Prx, peroxiredoxin; SelD, selenophosphate synthetase; SelW-like, selenoprotein W-like; FdhA, formate dehydrogenase alpha subunit; Trx-like, thioredoxin-like.

In addition to latitude/temperature, many other environmental factors may contribute to the evolution of metalloprotein and/or selenoprotein genes. We investigated the relationship between a variety of environmental features and metalloprotein or selenoprotein families based on canonical correlation analysis (CCA) and further generated the relevance network. Figure 5A–E shows significant correlations for each trace element, involving a total of 14 environmental factors and 27 protein families (significant association scores are shown in Table S7; the cutoff is set to 0.3; see Materials and methods). Both positive and negative correlations were observed for all of these elements except Se, suggesting a complex relationship between environmental stressors and trace element-dependent proteins. Eighteen families were found to be significantly correlated with multiple factors, especially SO (8 variables), NiSOD (8 variables), NiR (5 variables), DMSOR (5 variables), and urease (5 variables). Besides temperature which is correlated with all examined elements as expected, additional environmental factors were found to be correlated with specific proteins for multiple elements, such as solar insolation (for Cu, Mo, Ni, and Co), ocean acidification (for Mo, Ni, and Se), and sample depth and nitrate concentration (both for Cu, Mo, and Se). The top 5 factors correlating with multiple protein families include temperature (15 protein families), sample depth (10 protein families), solar insolation (7 protein families), nitrate concentration (7 protein families), and ocean acidification (7 protein families). Some of our findings are consistent with previous observations or hypotheses. For example, it has been reported that increased temperature could promote the production of urease in marine bacteria, suggesting a positive correlation between urease and temperature [45]. NiR might be positively correlated with nitrate level as it directly participates in the reduction of nitrate, which is an important biogeochemical process in the global marine ecosystem [46]. Negative correlations were mainly observed between several human-related stressors (such as ocean pollution, shipping track, and fishing styles) and Ni-dependent NiSOD, implying that human activity inhibits the utilization of NiSOD, an important enzyme for protecting cells against oxidative stress, and therefore causes a threat to sea microbes. In a word, these results not only provide important clues to the evolutionary patterns of metalloprotein and selenoprotein genes in extant marine microbes but also help to understand how these organisms have adapted to or are affected by their local environments.

## Discussion

In the recent decade, there has been a growing interest in exploring marine microbial communities via large-scale metagenomic sequencing approaches, which may give us new insights into the abundance, diversity, and metabolic activities of microorganisms in diverse marine environments [47], [48], [49]. Characterization of the metaproteome could help to understand the organization and functions of the microbial world and its linkage with ecological systems. It is known that trace element abundances in modern oceans vary both laterally and vertically and often by large orders of magnitude [50], and bioavailability and utilization of these elements may greatly influence microbial activity in different sites of marine environments. Prediction of genes encoding trace element-dependent proteins in the marine metagenomic dataset may provide direct evidence for the roles of these micronutrients in microbial populations.

In this study, all shotgun sequencing reads were used to identify the GOS metalloproteomes (for Cu, Mo, Ni, and Co) and selenoproteomes. The reason why we chose these elements is that they are not only widely used by microbes but also characterized by proteins which are strictly dependent on the corresponding element. Our results show the importance of trace element utilization within the marine microbial world and provide a comprehensive analysis of metalloproteins and selenoproteins which are used by these organisms.

We generated the largest environmental metalloproteome and selenoproteome dataset, which contains more than 56,000 metalloprotein and selenoprotein gene sequences. The utilization of Cu, Ni, and Se is highly active in the tropical zone, whereas Co utilization is relatively restricted. In addition, non-marine aquatic environments appear to inhibit the utilization of Cu and Se, whereas the distance from the continent might influence the use of Ni. Analysis of the biogeographical distribution of metalloprotein and/or selenoprotein families reflects distinct activities of biological processes that these proteins are involved in. A striking finding is that 41.1% of the sequences of RNR II, the most abundant Co-dependent protein family detected in the GOS dataset, may originate from viral genomes (mostly bacteriophages). It has been reported that up to 70% of marine bacteria could be infected by phages [51]. These viruses may play a role in the biological and ecological processes of host bacteria through the expression of auxiliary metabolic genes (AMGs) during infection, thus, to influence the microbial diversity and biogeochemical cycling [52]. One of the most important AMGs is RNR, which is involved in nucleotide biosynthesis and has been detected in many viral genomes and metagenomes [52], [53]. We examined other metalloprotein and selenoprotein families and found that two cuproproteins, plastocyanin and Cu-Zn SOD, also have a significant portion of viral sequences (35.9% and 20.3%, respectively). This is consistent with previous observations that genes encoding the two proteins are present in many viral genomes including marine viruses [54], [55]. Other protein families examined in this study either lack or only have very few viral sequences in the GOS dataset.

An advantage of our study is that it not only demonstrates the utilization of individual trace elements but also provides valuable information about their interactions. Strong correlations between Cu and Ni, as well as Ni and Se, are the most prominent examples which are quite stable either across all samples or within different subgroups of samples. Moreover, a highly consistent trend was observed between Ni-dependent protein-rich/-poor and selenoprotein-rich/-poor samples, suggesting that the utilization of these two elements may be strongly correlated across the world oceans.

Analysis of the relationship between a set of environmental factors and metalloprpotein/selenoprotein families could help to identify genes whose evolution is influenced by these environmental features. SSC-env-based sample clustering showed a significant latitudinal trend, as previously observed. Analysis of the occurrence of each metalloprotein/selenoprotein family in tropical and temperate sample groups revealed that several families, especially the most abundant cuproproteins, Ni-dependent proteins, and selenoproteins, favored tropical conditions, which may contribute to the active utilization of corresponding elements in tropical sea area. Further CCA and relevance network analyses indicated that certain environmental factors could be significantly correlated with several metalloprotein and selenoprotein families. The most complex protein–environment interactions were observed for SO and NiSOD. With regard to environmental variables, temperature appeared to be the most important factor that might significantly influence at least 15 trace element-dependent proteins corresponding to all examined elements. Other protein families did not show a significant correlation with the environmental factors examined here, some of which might be involved in cellular processes that are independent from ocean habitats. It is also possible that additional environmental factors are correlated with the evolution of metalloprotein and/or selenoprotein genes. A future challenge would be to investigate the evolutionary trends of trace element utilization in other types of environments, such as terrestrial and host-associated habitats.

## Conclusion

In conclusion, we used metagenomic data from the GOS project to identify the metalloproteomes (for Cu, Mo, Ni, and Co) and selenoproteomes in a large number of marine microbial samples. Our analysis yielded the largest environmental metalloprotein and selenoprotein gene dataset reported to date. Moreover, interactions among different trace element utilization, as well as the relationships between metalloprotein/selenoprotein families and a variety of environmental factors, were also analyzed at much larger scales, which provide new insights into the complex and dynamic evolution of trace element utilization in marine microbial communities.

## Materials and methods

### Metagenomic sequences and other resources

The raw sequence datasets containing shotgun reads for 253 samples of the GOS survey were downloaded from NCBI (BioProject: PRJNA13694; https://www.ncbi.nlm.nih.gov/bioproject/?term=PRJNA13694) and EBI (ENA: PRJEB8968 and PRJEB10418; https://www.ebi.ac.uk/ena/browser/view/PRJEB8968, and https://www.ebi.ac.uk/ena/browser/view/PRJEB10418, respectively). We chose metagenomic data for organisms collected within 0.1–0.8 µm size range which was thought to be dominated by bacteria [25]. Samples containing less than 20,000 reads were excluded. The Sargasso Sea sample GS00A was also discarded because it has been suspected of contamination [56]. Finally, a total of 179 samples (14.3 billion nucleotides), which cover 158 distinct marine and 21 non-marine aquatic sites, were analyzed.

A total of 18 environmental features were collected in this study (details are shown in Table S1). First, all descriptive metadata available for GOS samples (including sample depth, temperature, salinity, and concentrations of dissolved oxygen, silicate, nitrate, and phosphate) were downloaded from the same websites at NCBI and EBI. To investigate the cumulative impacts of human and other stressors, raw stressor data (including demersal destructive fishing, demersal non-destructive high bycatch fishing, demersal non-destructive low bycatch fishing, pelagic low bycatch fishing, ocean acidification, ocean pollution, shipping track, sea surface temperature anomaly, and UV radiation) were downloaded from the Knowledge Network for Biocomplexity (KNB) Data Repository (https://knb.ecoinformatics.org/#view/doi:10.5063/F1S180FS) developed for facilitating ecological and environmental research [57], and selectively extracted for GOS samples according to coordinates using the Raster package (version 2.5-8) in R. These original stressor data were ln-transformed [ln (X + 1), X represents the original value] prior to further use as previously suggested [57]. Moreover, information about cloud fraction and solar insolation was retrieved from the NASA Earth Observations (NEO) System (https://neo.sci.gsfc.nasa.gov/) by using the average monthly values of aquatic locations closest to corresponding samples. The vectors of all environmental factors were Z-score transformed to allow direct comparison among factors with different units of measurement.

### Metagenome assembly

*De novo* assembly for metagenomes was performed with the Celera Assembler software (version 8.3) [58]. The parameters were defined as follows: utgErrorRate = 0.12, ovlErrorRate = 0.15, cnsErrorRate = 0.15, cgwErrorRate = 0.15, utgBubblePopping = 0, utgGenomeSize = 150,000, merSize = 14, doFragmentCorrection = 0, and doExtendClearRanges = 1. The assembly statistics is briefly shown in Table S8.

### Identification of metalloprotein and selenoprotein genes

In this study, metalloproteins refer to proteins that are strictly Me-binding proteins (Me represents a metal or metal-containing cofactor). Proteins that may bind alternative metals in different organisms were excluded. We collected a large number of known metalloproteins (for Cu, Mo, Ni, and Co) and selenoproteins from published resources [14], [15], [16], [17], [18], [19], [26]. In addition, literature searches were performed to include newly identified metalloproteins and selenoproteins. A list of known metalloprotein and selenoprotein families is shown in Table S9.

We used representative sequences of each metalloprotein family as seeds to search against each GOS sample for homologs via TBLASTN with default parameters. Distant homologs were further identified by using repetitive TBLASTN searches. A reasonable open reading frame (ORF) was predicted for each nucleotide sequence identified above. All protein sequences were then verified by examining the presence of conserved domains of corresponding metalloprotein families using various annotation databases such as COG, Pfam, TIGR, and CDD. Conserved metal-binding ligands or motifs were also examined to help identify metal-dependent forms of metalloprotein families [17].

With regard to selenoproteins, we adopted an approach that was previously used for the identification of selenoproteins in genomic datasets [19], [26]. Briefly, representative sequences of each bacterial selenoprotein family were used to search against the GOS dataset for selenoprotein homologs via TBLASTN with default parameters. The Sec/UGA pairs were selected, and the ORF constraint was examined for each UGA-containing nucleotide sequence. Redundant selenoprotein sequences were removed, and the presence of a possible Sec insertion sequence (SECIS) element downstream of the Sec-encoding UGA codon was analyzed for questionable sequences using bSECISearch program [59]. Considering that almost all selenoproteins have homologs in which Sec is replaced by Cys [40], all remaining sequences were further searched against the NCBI non-redundant protein database for the presence of conserved Cys-containing homologs via BLASTP.

The fraction of genes encoding metalloproteins or selenoproteins in each sample was normalized using the number of reads covering the corresponding genes divided by the total number of reads obtained for the sample. Using the same criteria that were previously employed to evaluate the abundance of selenoprotein genes in part of the GOS dataset [26], we designated samples as metalloprotein- or selenoprotein-rich samples if they contained at least 1.5 times the average level, and metalloprotein- or selenoprotein-poor samples if they had no more than half the average level of metalloproteins or selenoproteins.

### Correlation analysis

To investigate the relationships between environmental features and GOS samples, we adopted a similar strategy that has been successfully used for analyzing environmental adaptation of metabolic pathways and membrane proteins in a subset of GOS samples [41], [42]. Based on the normalized values of all environmental features, an environmental feature matrix was built where the rows and columns represent samples and environmental variables, respectively. Pairwise Spearman correlation analysis was performed to study the SSC on the basis of environmental variables (SSC-env). Hierarchical clustering and heatmap analysis were performed using the heatmap.2 function from gplots package of R.

CCA was performed to assess the relationship between environmental features and metalloprotein/selenoprotein families by using the mixOmics software package (version 6.1.3) [60] in R. Protein families that were detected in less than 10 GOS samples were ignored. Based on the CCA-derived pairwise similarity matrix in which values (association scores) could be considered as a robust representation of the correlation [61], we constructed the relevance network using the *network* function in the mixOmics package. In such a network, each edge represents the association of two corresponding nodes (environmental variables and protein families). To highlight the strongest associations and to obtain biologically interpretable networks, only values exceeding a specified threshold (0.3 as defined here) were considered as significant associations. Finally, the obtained networks were presented using Cytoscape software (version 3.5.1) for visualization [62].

## Code availability

The scripts used for the aforementioned analyses are publicly accessible at https://github.com/Janetis/Trace_Element_Utilization.

## Competing interests

The authors have declared no competing interests.

## CRediT authorship contribution statement

**Yinzhen Xu:** Methodology, Software, Resources, Data curation, Formal analysis, Visualization, Writing – original draft, Writing – review & editing. **Jiayu Cao:** Resources, Data curation, Writing – review & editing. **Liang Jiang:** Supervision, Writing – review & editing. **Yan Zhang:** Conceptualization, Supervision, Project administration, Funding acquisition, Writing – review & editing.

## Supplementary material

Supplementary File S1 **The file containing all metalloprotein and selenoprotein sequences predicted in this study**

Supplementary Figure S1 **A diagram of the workflow for comparative analysis of trace element utilization in GOS samples.**

Supplementary Figure S2 **The occurrence of cuproproteomes in GOS samples** The boundary lines for cuproprotein-rich and -poor samples are highlighted. Sequential color schemes are used to show different geographical areas. Tropical and temperate regions are also shown. The sample containing the largest (normalized) fraction of cuproprotein genes is indicated.

Supplementary Figure S3 **The occurrence of molybdoproteomes in GOS samples** The boundary lines for molybdoprotein-rich and -poor samples are highlighted. The sample containing the largest (normalized) fraction of molybdoprotein genes is indicated.

Supplementary Figure S4 **The occurrence of Ni-dependent proteomes in GOS samples** The boundary lines for Ni-dependent-protein-rich and -poor samples are highlighted. The sample containing the largest (normalized) fraction of Ni-dependent protein genes is indicated.

Supplementary Figure S5 **The occurrence of Co-dependent proteomes in GOS samples** The boundary lines for Co-dependent-protein-rich and -poor samples are highlighted. The sample containing the largest (normalized) fraction of Co-dependent protein genes is indicated.

Supplementary Figure S6 **Putative taxonomic affiliation of RNR II sequences detected in the GOS dataset**

Supplementary Figure S7 **The occurrence of selenoproteomes in GOS samples** The boundary lines for selenoprotein-rich and -poor samples are highlighted. The sample containing the largest (normalized) fraction of selenoprotein genes is indicated.

Supplementary Figure S8 **Spearman rank correlation and clustering analyses of GOS samples based on environmental features** Each sample is defined by a vector of 18 environmental variables. The sample pairs are color-coded according to their similarity.
