# Supplementary material for: Biogeographic and Evolutionary Patterns of Trace Element Utilization in Marine Microbial World
Source: Genomics Proteomics Bioinformatics. 2021 Feb 23;19(6):958–72. doi: 10.1016/j.gpb.2021.02.003 (PMC9402790; doi:10.1016/j.gpb.2021.02.003)
Supplement: Supplementary Figure S1 — A diagram of the workflow for comparative analysis of trace element utilization in GOS samples. [file mmc2.pdf]

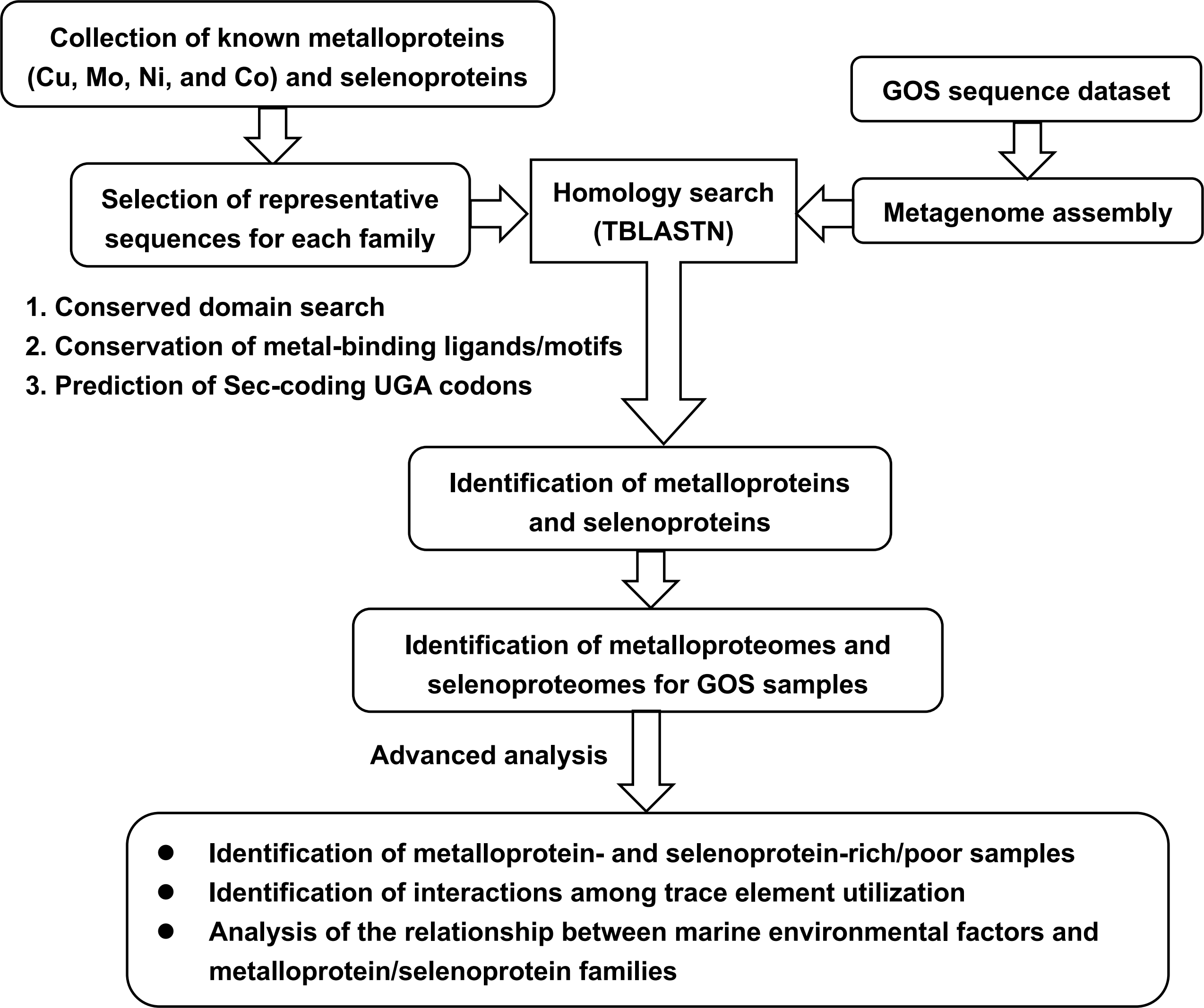
Collection of known metalloproteins (Cu, Mo, Ni, and Co) and selenoproteins
GOS sequence dataset
Selection of representative sequences for each family
Homology search (TBLASTN)
Metagenome assembly
1. Conserved domain search
2. Conservation of metal-binding ligands/motifs
3. Prediction of Sec-coding UGA codons
Identification of metalloproteins and selenoproteins
Identification of metalloproteomes and selenoproteomes for GOS samples
Advanced analysis
Identification of metalloprotein- and selenoprotein-rich/poor samples
Identification of interactions among trace element utilization
Analysis of the relationship between marine environmental factors and metalloprotein/selenoprotein families
