# Supplementary figures and images for: Biogeographic and Evolutionary Patterns of Trace Element Utilization in Marine Microbial World
Source: Genomics Proteomics Bioinformatics. 2021 Feb 23;19(6):958–72. doi: 10.1016/j.gpb.2021.02.003 (PMC9402790; doi:10.1016/j.gpb.2021.02.003)

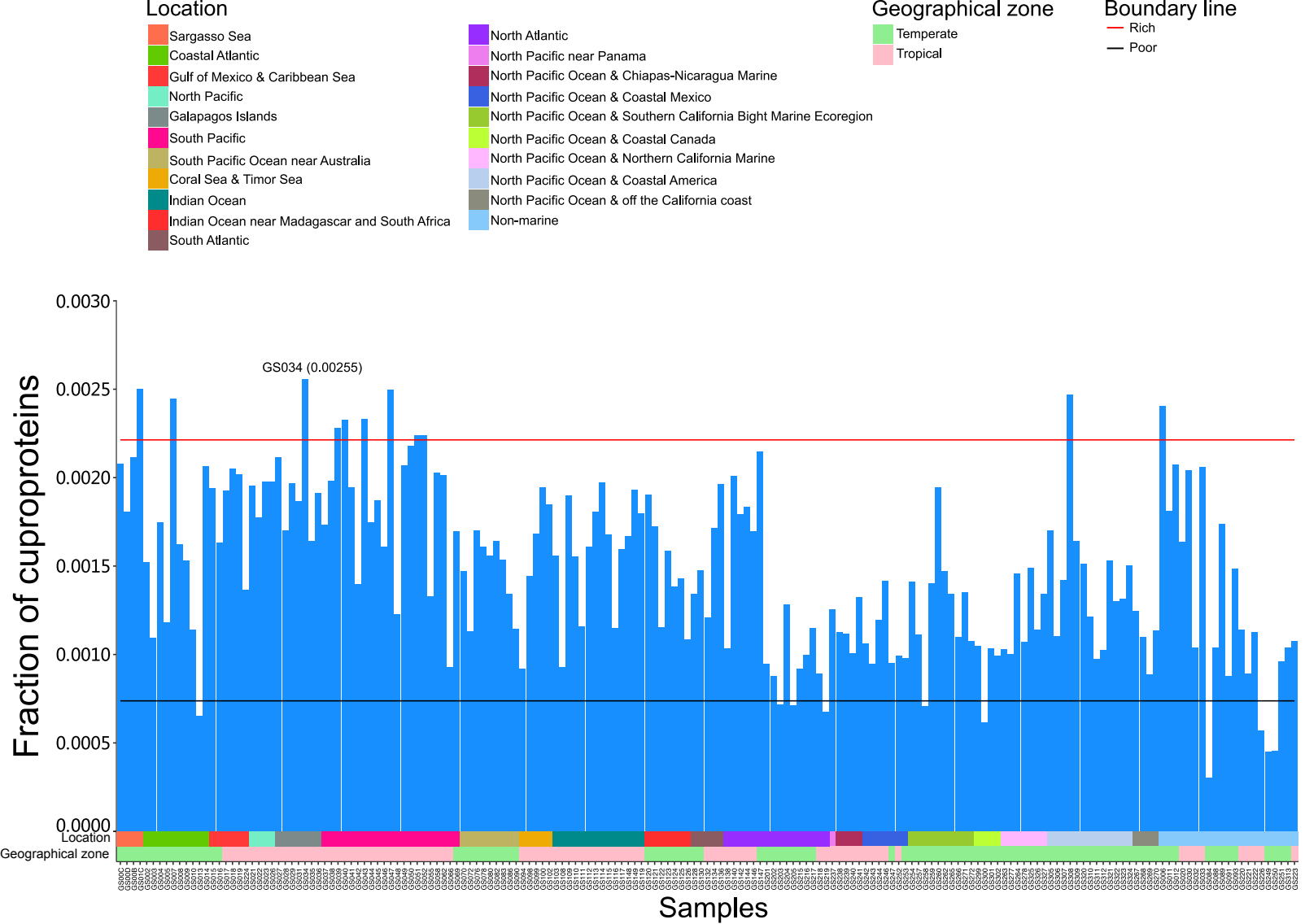

Supplement: Supplementary Figure S2 — The occurrence of cuproproteomes in GOS samples The boundary lines for cuproprotein-rich and -poor samples are highlighted. Sequential color schemes are used to show different geographical areas. Tropical and temperate regions are also shown. The sample containing the largest (normalized) fraction of cuproprotein genes is indicated. [file mmc3.pdf]

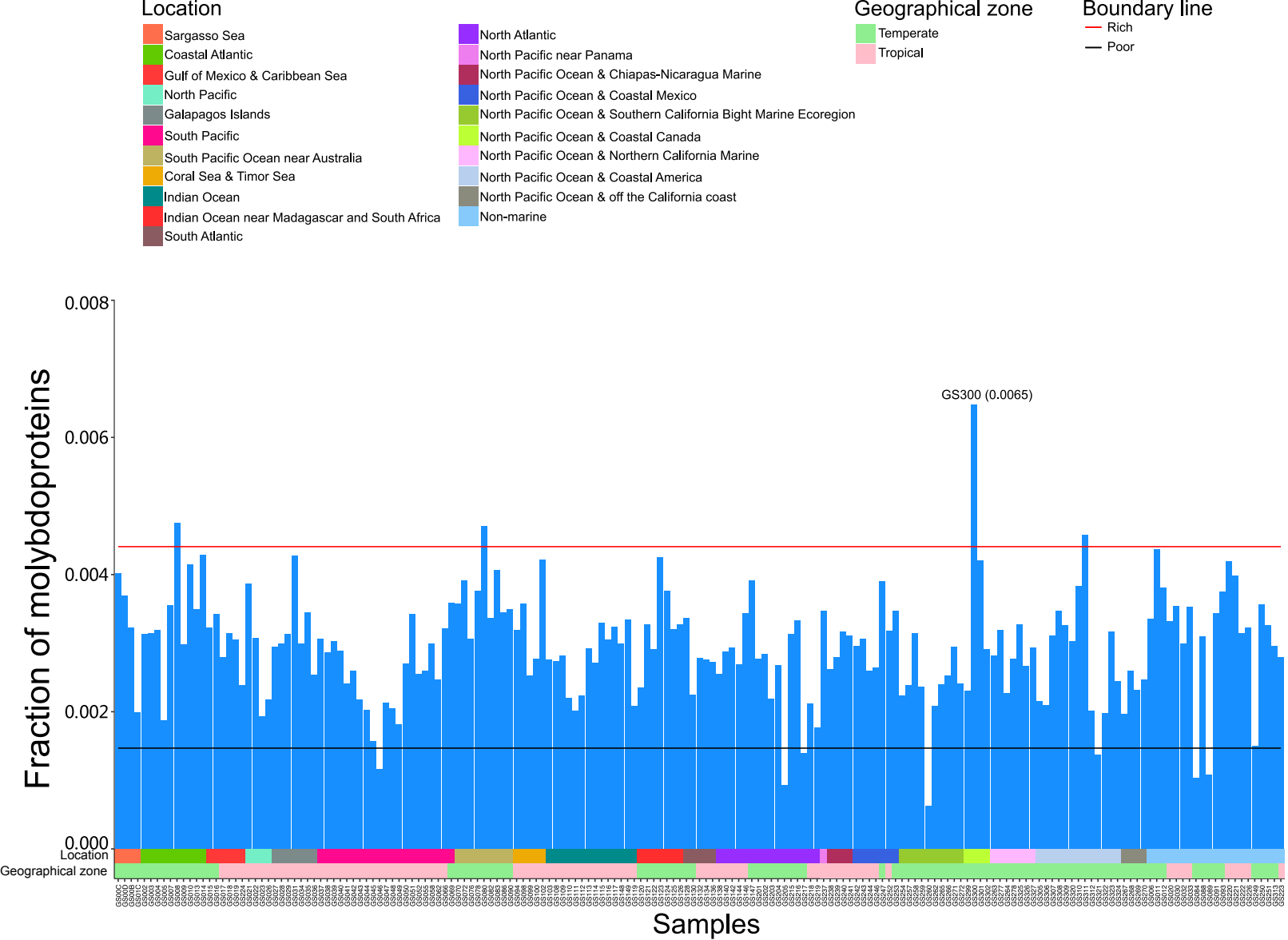

Supplement: Supplementary Figure S3 — The occurrence of molybdoproteomes in GOS samples The boundary lines for molybdoprotein-rich and -poor samples are highlighted. The sample containing the largest (normalized) fraction of molybdoprotein genes is indicated. [file mmc4.pdf]

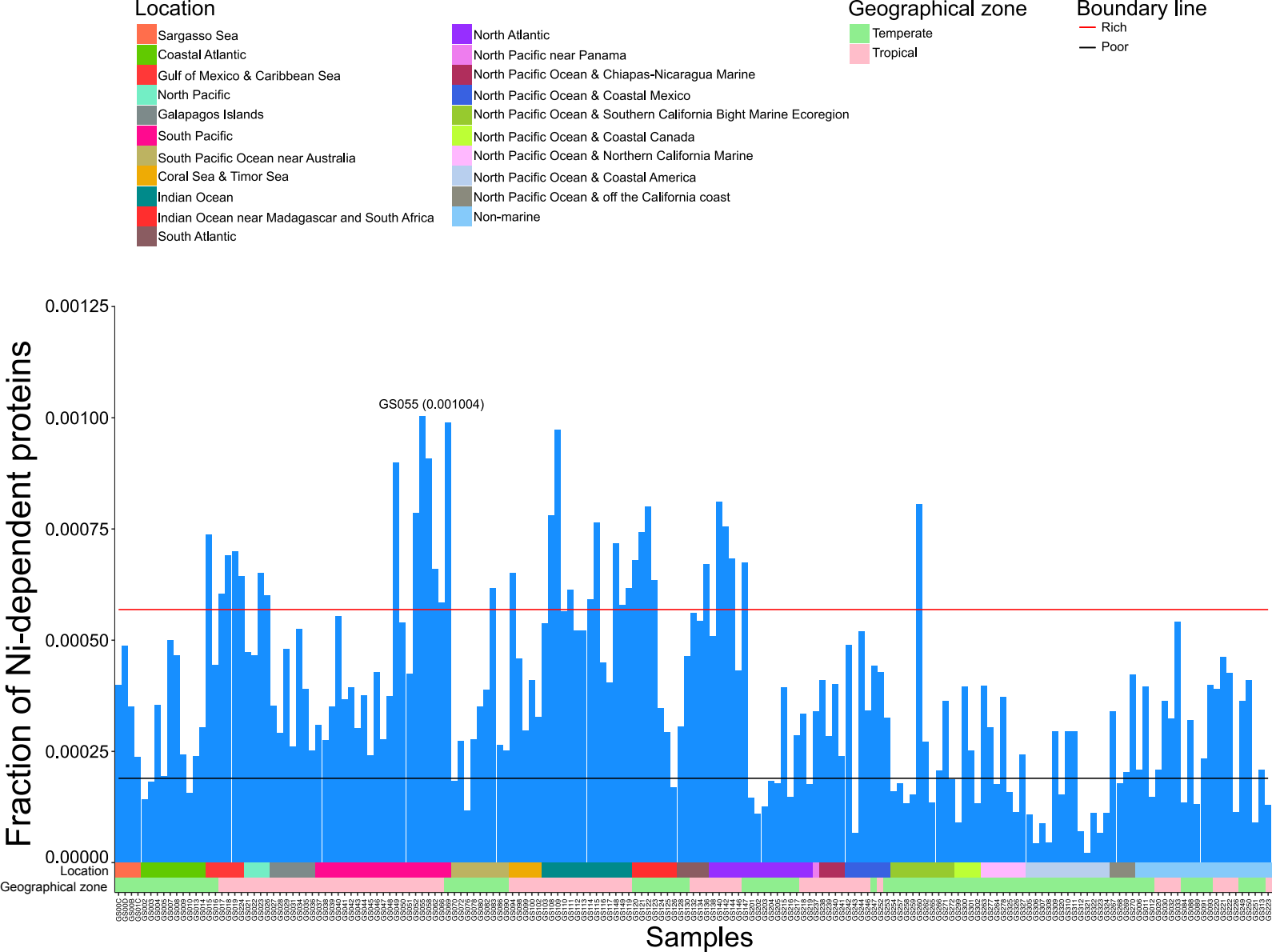

Supplement: Supplementary Figure S4 — The occurrence of Ni-dependent proteomes in GOS samples The boundary lines for Ni-dependent-protein-rich and -poor samples are highlighted. The sample containing the largest (normalized) fraction of Ni-dependent protein genes is indicated. [file mmc5.pdf]

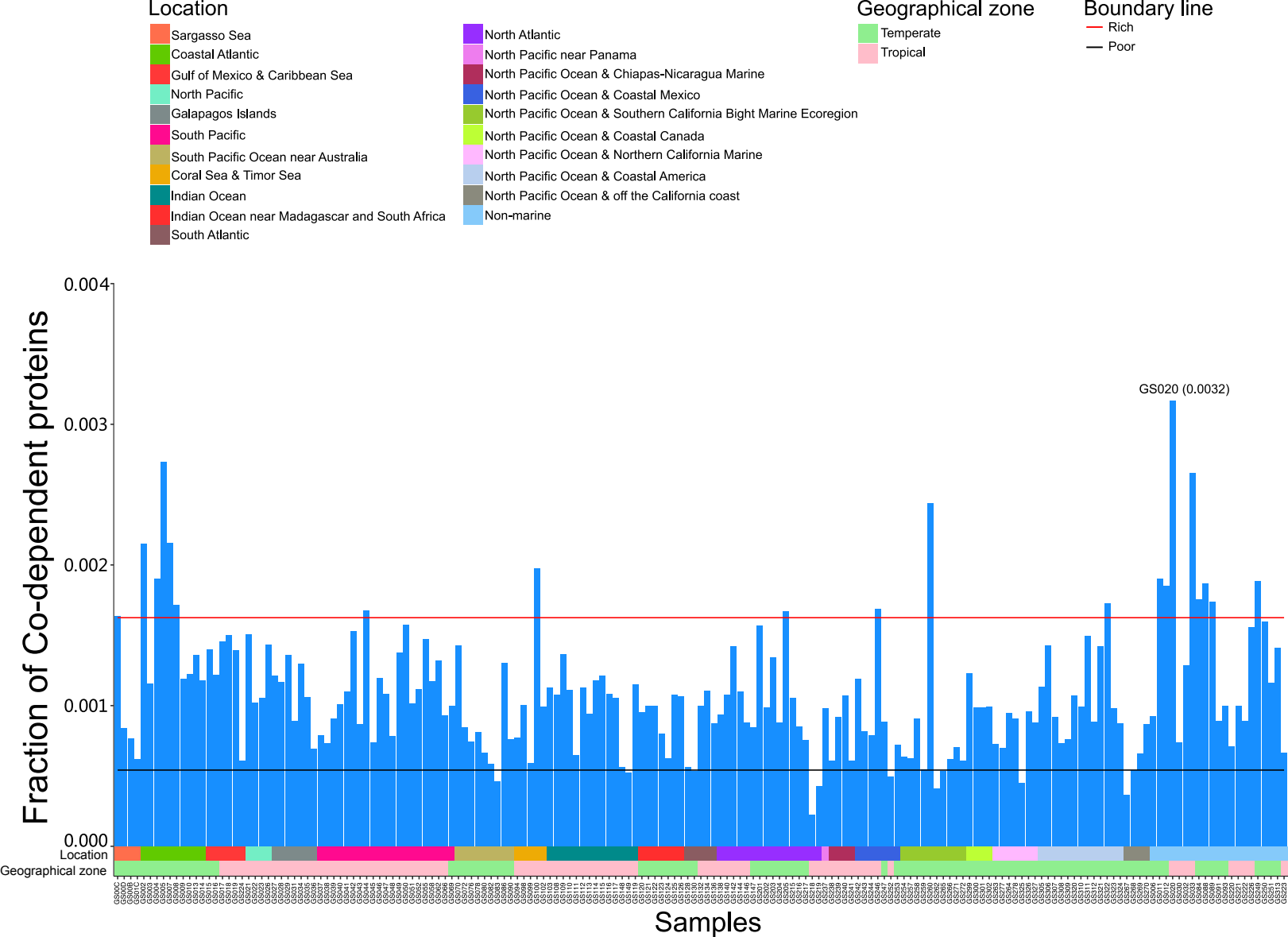

Supplement: Supplementary Figure S5 — The occurrence of Co-dependent proteomes in GOS samples The boundary lines for Co-dependent-protein-rich and -poor samples are highlighted. The sample containing the largest (normalized) fraction of Co-dependent protein genes is indicated. [file mmc6.pdf]

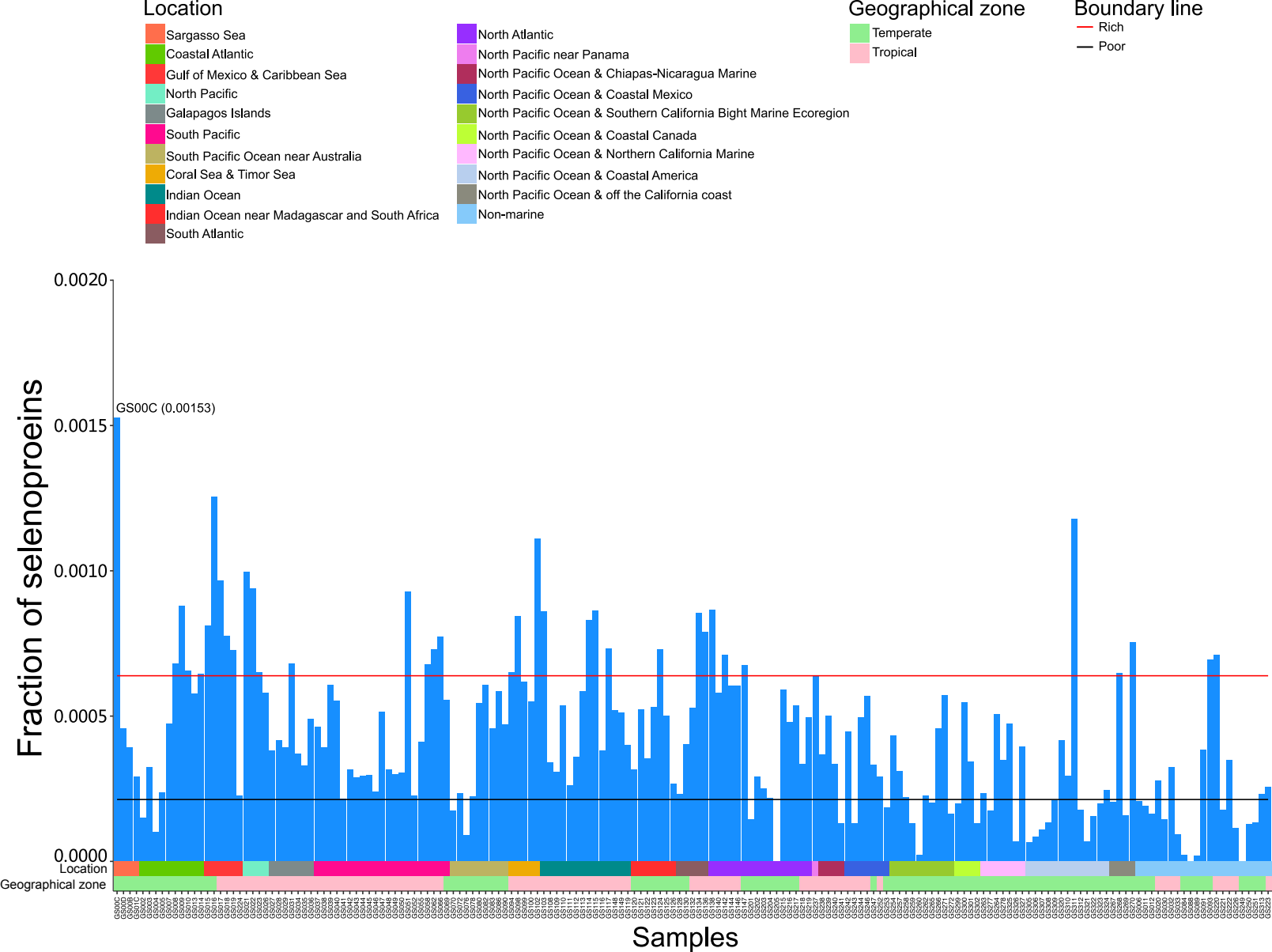

Supplement: Supplementary Figure S7 — The occurrence of selenoproteomes in GOS samples The boundary lines for selenoprotein-rich and -poor samples are highlighted. The sample containing the largest (normalized) fraction of selenoprotein genes is indicated. [file mmc8.pdf]

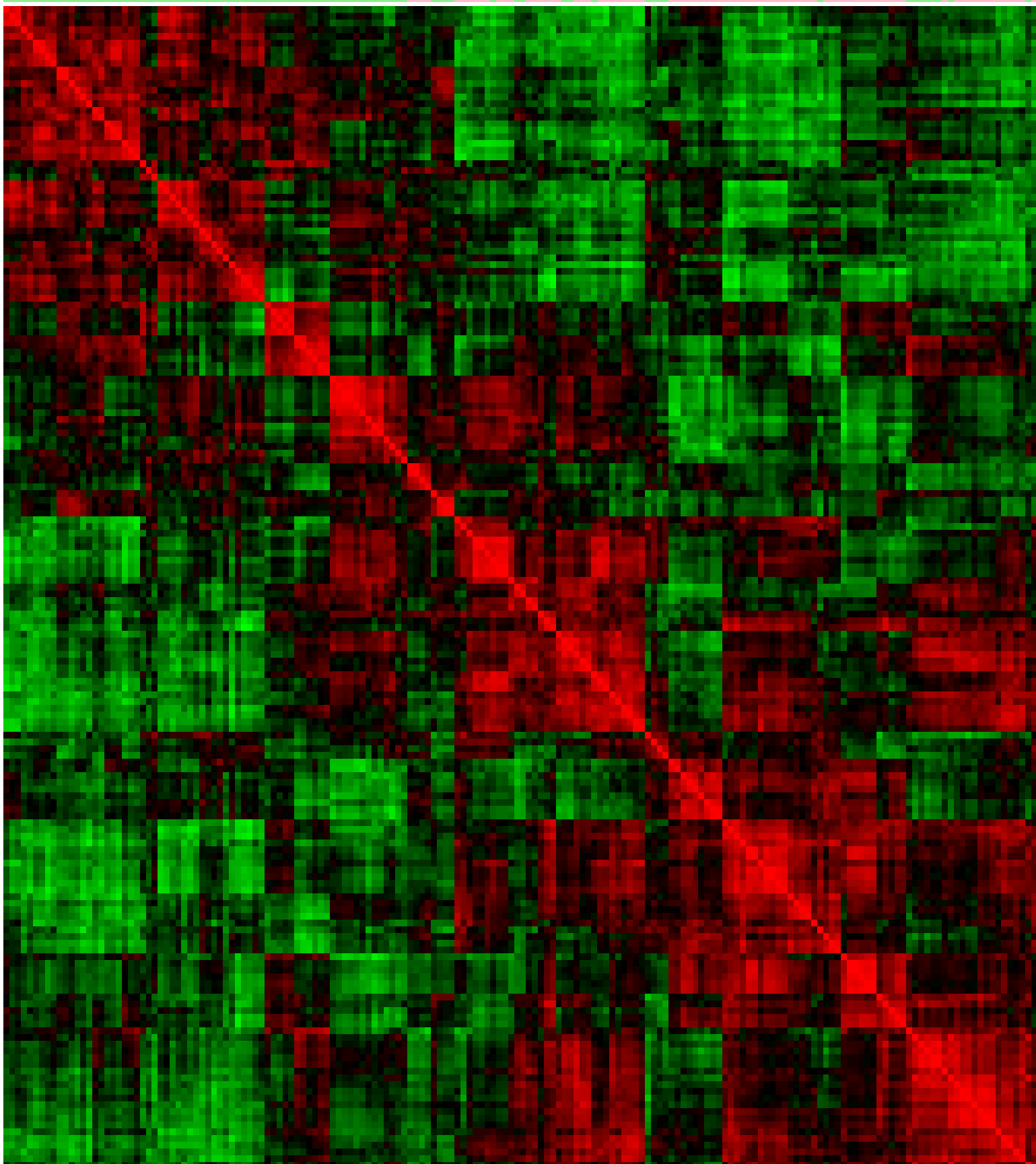

Spearman correlation coefficient

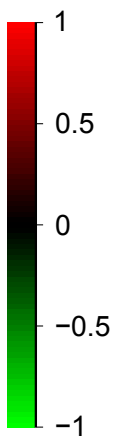

Geographical zone

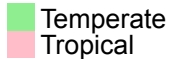

Supplement: Supplementary Figure S8 — Spearman rank correlation and clustering analyses of GOS samples based on environmental features Each sample is defined by a vector of 18 environmental variables. The sample pairs are color-coded according to their similarity. [file mmc9.pdf]
