# Supplementary material for: Biogeographic and Evolutionary Patterns of Trace Element Utilization in Marine Microbial World
Source: Genomics Proteomics Bioinformatics. 2021 Feb 23;19(6):958–72. doi: 10.1016/j.gpb.2021.02.003 (PMC9402790; doi:10.1016/j.gpb.2021.02.003)
Supplement: Supplementary Figure S6 — Putative taxonomic affiliation of RNR II sequences detected in the GOS dataset [file mmc7.pdf]

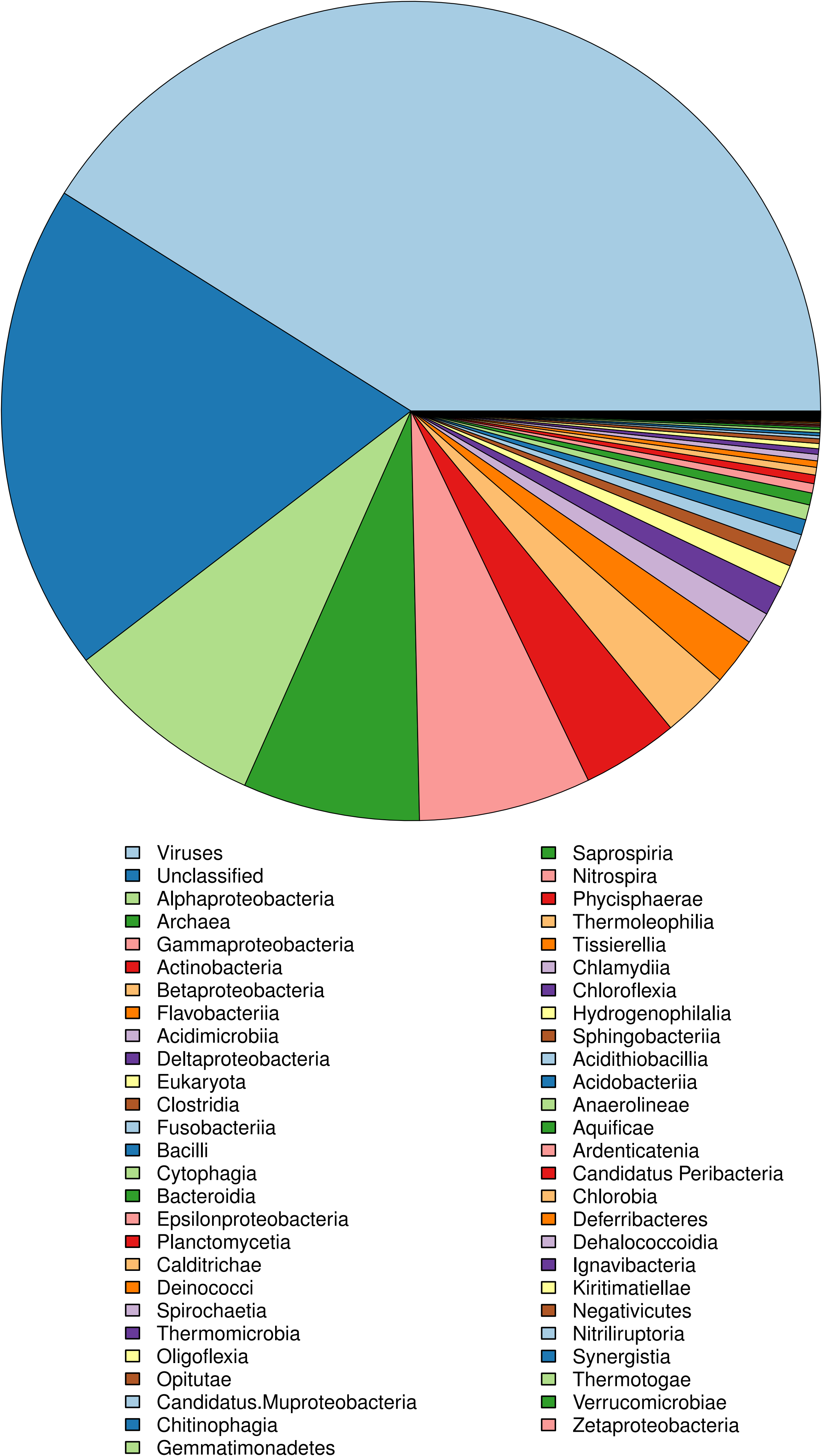
Viruses
Unclassified
Alphaproteobacteria
Archaea
Gammaproteobacteria
Actinobacteria
Betaproteobacteria
Flavobacteriia
Acidimicrobiia
Deltaproteobacteria
Eukaryota
Clostridia
Fusobacteriia
Bacilli
Cytophagia
Bacteroidia
Epsilonproteobacteria
Planctomycetia
Calditrichae
Deinococci
Spirochaetia
Thermomicrobia
Oligoflexia
Opitutae
Candidatus.Muproteobacteria
Chitinophagia
Gemmatimonadetes
Saprospiria
Nitrospira
Phycisphaerae
Thermoleophilia
Tissierellia
Chlamydiia
Chloroflexia
Hydrogenophilalia
Sphingobacteriia
Acidithiobacillia
Acidobacteriia
Anaerolineae
Aquificae
Ardenticatenia
Candidatus Peribacteria
Chlorobia
Deferribacteres
Dehalococcoidia
Ignavibacteria
Kiritimatiellae
Negativicutes
Nitriliruptoria
Synergistia
Thermotogae
Verrucomicrobiae
Zetaproteobacteria
