# Supplementary material for: Biogeographic and Evolutionary Patterns of Trace Element Utilization in Marine Microbial World
Source: Genomics Proteomics Bioinformatics. 2021 Feb 23;19(6):958–72. doi: 10.1016/j.gpb.2021.02.003 (PMC9402790; doi:10.1016/j.gpb.2021.02.003)
Supplement: Supplementary Table S7 [file mmc16.docx]

**Table S7 Significant association scores for the correlation between environmental factors and protein families**

| **Trace element** | **Protein family** | **Demersal destructive fishing** | **Demersal nondestructive high bycatch** | **Demersal nondestructive low bycatch** | **Sample depth** | **Dissolved oxygen** | **Nitrate** | **Ocean acidification** | **Ocean pollution** | **Phosphate** | **Shipping tracks** | **Silicate** | **Solar insolation** | **Temperature** | **UV radiation** |
| --- | --- | --- | --- | --- | --- | --- | --- | --- | --- | --- | --- | --- | --- | --- | --- |
| Cu | COX I | - | - | - | - | - | - | - | - | - | - | - | 0.33 | 0.39 | - |
|  | COX II | - | - | - | - | - | - | - | - | - | - | - | 0.39 | 0.47 | - |
|  | Nitrite reductase | - | - | - | 0.79 | - | 0.77 | - | - | 0.64 | - | 0.54 | - | −0.35 | - |
|  | Plastocyanin | - | - | - | - | - | - | - | - | - | - | - | - | 0.35 | - |
|  | Cu-Zn superoxide dismutase | - | - | - | - | - | - | - | - | - | - | - | −0.32 | −0.34 | - |
|  | pMMO | - | - | - | 0.41 | - | 0.41 | - | - | 0.37 | - | - | - | - | - |
| Mo | SO | 0.41 | 0.33 | 0.35 | - | - | - | −0.32 | 0.42 | - | - | - | −0.32 | −0.44 | 0.36 |
|  | XO | - | - | - | 0.33 | - | - | 0.33 | - | - | - | - | - | - | - |
|  | DMSOR | 0.33 | - | - | - | - | 0.36 | - | 0.42 | - | 0.3 | - | - | −0.38 | - |
| Ni | Urease | - | - | - | - | −0.33 | - | 0.5 | −0.35 | - | - | - | 0.45 | 0.55 | - |
|  | NiSOD | −0.31 | - | −0.3 | - | - | - | 0.37 | −0.54 | −0.31 | −0.3 | - | 0.6 | 0.53 | - |
| Co | EAL | - | - | - | - | - | - | - | - | - | - | 0.31 | - | - | - |
|  | LitR/CarH/CarA | - | - | - | - | - | - | - | - | - | - | - | - | −0.31 | - |
|  | MCM | - | - | - | - | - | - | - | - | - | - | - | - | 0.3 | - |
|  | MetH | - | - | - | - | - | - | - | - | - | - | - | - | 0.34 | - |
|  | PpaA | - | - | - | - | - | - | - | - | - | - | - | 0.34 | 0.4 | - |
|  | RNR II | 0.35 | - | 0.42 | - | - | - | - | - | - | - | 0.48 | - | - | - |
| Se | Arsenate reductase | - | - | - | 0.52 | - | 0.33 | - | - | - | - | - | - | - | - |
|  | Deiodinase-like | - | - | - | 0.57 | - | 0.42 | - | - | 0.35 | - | - | - | - | - |
|  | DsbA-like | - | - | - | 0.71 | - | 0.5 | - | - | 0.41 | - | 0.31 | - | - | - |
|  | FdhA | - | - | - | 0.33 | - | - | - | - | - | - | - | - | - | - |
|  | Hypothetical protein | - | - | - | 0.31 | - | - | - | - | - | - | - | - | - | - |
|  | Peroxiredoxin | - | - | - | 0.33 | - | - | 0.33 | - | - | - | - | - | - | - |
|  | SelD | - | - | - | - | - | - | 0.32 | - | - | - | - | - | 0.36 | - |
|  | SelW-like | - | - | - | - | - | - | 0.34 | - | - | - | - | - | - | - |
|  | Trx-like | - | - | - | - | - | - | - | - | - | - | - | - | 0.32 | - |
|  | ULPU-containing | - | - | - | 0.5 | - | 0.36 | - | - | - | - | - | - | - | - |
