# Supplementary material for: Biogeographic and Evolutionary Patterns of Trace Element Utilization in Marine Microbial World
Source: Genomics Proteomics Bioinformatics. 2021 Feb 23;19(6):958–72. doi: 10.1016/j.gpb.2021.02.003 (PMC9402790; doi:10.1016/j.gpb.2021.02.003)
Supplement: Supplementary Table S8 [file mmc17.docx]

**Table S8 Assembly statistics for GOS metagenomes**

| **Sample ID** | **Base count** | **Read count** | **No. of assembled sequences** |
| --- | --- | --- | --- |
| GS00B | 320,788,711 | 316,918 | 176,489 |
| GS00C | 371,688,990 | 368,835 | 180,933 |
| GS00D | 336,156,351 | 332,135 | 200,011 |
| GS01C | 92,688,958 | 92,351 | 52,844 |
| GS002 | 90,323,273 | 105,994 | 41,297 |
| GS003 | 41,750,462 | 49,370 | 34,819 |
| GS004 | 33,287,155 | 39,466 | 18,591 |
| GS005 | 39,582,872 | 46,522 | 21,546 |
| GS006 | 36,602,019 | 43,230 | 24,924 |
| GS007 | 32,428,379 | 38,006 | 17,451 |
| GS008 | 103,145,875 | 120,280 | 55,752 |
| GS009 | 61,474,474 | 73,864 | 31,386 |
| GS010 | 59,264,513 | 70,170 | 47,173 |
| GS011 | 100,211,326 | 121,084 | 55,807 |
| GS012 | 103,549,464 | 122,642 | 48,668 |
| GS013 | 104,976,966 | 133,268 | 97,373 |
| GS014 | 92,065,606 | 108,458 | 60,552 |
| GS015 | 89,638,650 | 107,156 | 50,557 |
| GS016 | 88,924,874 | 105,988 | 56,758 |
| GS017 | 200,973,665 | 240,216 | 134,158 |
| GS018 | 120,792,414 | 140,616 | 85,073 |
| GS019 | 93,575,098 | 111,460 | 48,231 |
| GS020 | 216,450,149 | 269,210 | 114,159 |
| GS021 | 93,854,931 | 114,240 | 56,771 |
| GS022 | 84,580,893 | 103,198 | 46,081 |
| GS023 | 104,989,753 | 128,962 | 74,349 |
| GS026 | 77,631,287 | 98,274 | 39,531 |
| GS027 | 177,309,056 | 215,272 | 122,298 |
| GS028 | 154,093,353 | 184,882 | 103,295 |
| GS029 | 103,966,672 | 127,166 | 50,290 |
| GS030 | 286,002,928 | 346,912 | 106,570 |
| GS031 | 342,315,733 | 422,294 | 186,645 |
| GS032 | 117,986,382 | 138,704 | 61,376 |
| GS033 | 580,089,290 | 709,382 | 111,896 |
| GS034 | 101,701,190 | 129,552 | 51,944 |
| GS035 | 111,543,878 | 136,042 | 58,288 |
| GS036 | 63,039,734 | 75,400 | 50,771 |
| GS037 | 55,331,210 | 64,700 | 26,227 |
| GS038 | 125,322,934 | 145,408 | 51,483 |
| GS039 | 84,244,724 | 93,880 | 45,534 |
| GS040 | 80,148,086 | 90,360 | 42,290 |
| GS041 | 82,821,276 | 92,606 | 37,215 |
| GS042 | 81,953,169 | 91,542 | 39,757 |
| GS043 | 75,882,808 | 86,224 | 42,934 |
| GS044 | 70,820,167 | 85,232 | 43,125 |
| GS045 | 70,445,376 | 87,058 | 50,526 |
| GS046 | 63,886,498 | 74,624 | 31,687 |
| GS047 | 138,025,866 | 159,210 | 77,385 |
| GS048 | 69,811,897 | 88,138 | 65,245 |
| GS049 | 72,948,572 | 90,014 | 51,361 |
| GS050 | 74,885,937 | 94,656 | 41,470 |
| GS051 | 169,842,222 | 206,878 | 91,072 |
| GS052 | 48,487,298 | 57,212 | 36,384 |
| GS055 | 46,135,187 | 55,788 | 36,704 |
| GS058 | 139,979,355 | 175,182 | 99,712 |
| GS062 | 48,548,604 | 57,608 | 38,307 |
| GS066 | 46,897,810 | 58,124 | 44,440 |
| GS069 | 118,821,158 | 149,616 | 81,198 |
| GS070 | 89,080,117 | 108,842 | 62,990 |
| GS072 | 65,529,817 | 80,634 | 55,821 |
| GS076 | 121,962,030 | 153,378 | 96,626 |
| GS078 | 70,031,495 | 93,786 | 60,761 |
| GS080 | 80,635,354 | 102,770 | 57,338 |
| GS082 | 111,439,719 | 136,450 | 86,130 |
| GS083 | 48,578,955 | 56,654 | 41,490 |
| GS084 | 205,581,097 | 274,064 | 126,070 |
| GS086 | 84,172,394 | 106,004 | 65,576 |
| GS088 | 205,281,168 | 249,360 | 62,747 |
| GS089 | 204,399,989 | 258,346 | 82,437 |
| GS090 | 105,119,503 | 131,116 | 90,290 |
| GS091 | 85,332,022 | 106,994 | 73,995 |
| GS093 | 166,015,143 | 205,754 | 117,907 |
| GS094 | 48,691,273 | 59,844 | 44,464 |
| GS098 | 47,567,577 | 56,780 | 41,670 |
| GS099 | 121,744,819 | 155,146 | 91,164 |
| GS100 | 206,288,378 | 261,244 | 97,940 |
| GS102 | 128,866,267 | 158,470 | 89,351 |
| GS103 | 44,315,608 | 55,856 | 40,744 |
| GS108 | 44,325,490 | 61,448 | 42,787 |
| GS109 | 51,258,256 | 58,558 | 40,439 |
| GS110 | 76,023,538 | 108,164 | 71,305 |
| GS111 | 48,055,302 | 57,136 | 43,064 |
| GS112 | 75,180,631 | 105,592 | 71,993 |
| GS113 | 92,278,761 | 107,328 | 69,457 |
| GS114 | 275,729,142 | 352,896 | 184,654 |
| GS115 | 52,078,059 | 60,166 | 42,017 |
| GS116 | 49,678,443 | 60,082 | 45,016 |
| GS117 | 260,424,058 | 348,008 | 190,843 |
| GS119 | 52,042,355 | 60,050 | 41,800 |
| GS120 | 33,902,535 | 44,176 | 31,148 |
| GS121 | 93,723,280 | 109,076 | 69,677 |
| GS122 | 82,335,893 | 110,044 | 78,507 |
| GS123 | 87,229,441 | 105,380 | 77,911 |
| GS124 | 91,826,164 | 115,080 | 79,244 |
| GS125 | 196,578,946 | 248,854 | 146,064 |
| GS126 | 138,873,978 | 165,282 | 98,026 |
| GS128 | 116,280,783 | 150,714 | 96,925 |
| GS130 | 79,511,837 | 99,024 | 68,011 |
| GS132 | 124,167,889 | 151,260 | 100,100 |
| GS134 | 91,840,098 | 108,602 | 67,047 |
| GS136 | 48,376,855 | 59,556 | 41,887 |
| GS138 | 47,871,540 | 58,970 | 44,227 |
| GS140 | 46,172,662 | 56,762 | 37,317 |
| GS142 | 54,835,660 | 67,564 | 43,989 |
| GS144 | 83,822,792 | 100,998 | 61,054 |
| GS146 | 47,003,708 | 57,872 | 42,033 |
| GS147 | 67,750,610 | 81,552 | 54,805 |
| GS148 | 81,669,444 | 101,828 | 72,888 |
| GS149 | 86,111,464 | 107,150 | 69,381 |
| GS201 | 20,372,202 | 27,446 | 23,330 |
| GS202 | 20,381,304 | 27,438 | 20,696 |
| GS203 | 17,973,976 | 23,814 | 19,103 |
| GS204 | 19,611,438 | 27,340 | 20,947 |
| GS205 | 21,830,566 | 28,146 | 20,342 |
| GS215 | 37,149,210 | 45,636 | 37,981 |
| GS216 | 21,046,085 | 27,048 | 23,549 |
| GS217 | 20,391,961 | 27,894 | 24,208 |
| GS218 | 20,800,808 | 26,896 | 22,036 |
| GS219 | 22,559,938 | 28,218 | 21,236 |
| GS220 | 21,317,496 | 28,132 | 22,465 |
| GS221 | 21,686,279 | 28,106 | 23,441 |
| GS222 | 20,544,346 | 25,798 | 19,721 |
| GS223 | 37,533,018 | 46,544 | 33,288 |
| GS224 | 20,399,760 | 26,406 | 20,207 |
| GS226 | 18,788,845 | 26,368 | 22,182 |
| GS237 | 35,617,038 | 47,056 | 36,592 |
| GS238 | 37,516,023 | 46,272 | 36,655 |
| GS239 | 36,400,772 | 45,758 | 35,934 |
| GS240 | 35,310,705 | 44,818 | 35,287 |
| GS241 | 37,235,594 | 46,104 | 31,702 |
| GS242 | 36,510,545 | 47,056 | 34,827 |
| GS243 | 36,612,374 | 45,412 | 35,040 |
| GS244 | 35,485,775 | 44,314 | 34,989 |
| GS246 | 36,803,577 | 43,874 | 33,808 |
| GS247 | 35,291,568 | 45,198 | 34,444 |
| GS249 | 35,136,946 | 46,748 | 28,418 |
| GS250 | 37,309,743 | 46,408 | 34,970 |
| GS251 | 35,331,858 | 44,796 | 24,280 |
| GS252 | 34,519,735 | 44,396 | 37,134 |
| GS253 | 33,831,903 | 42,968 | 34,725 |
| GS254 | 34,583,737 | 43,900 | 34,476 |
| GS257 | 34,771,903 | 44,916 | 33,143 |
| GS258 | 35,729,650 | 45,162 | 35,528 |
| GS259 | 36,458,618 | 45,676 | 36,394 |
| GS260 | 32,106,441 | 44,720 | 6392 |
| GS262 | 34,725,925 | 44,218 | 34,613 |
| GS263 | 33,289,706 | 42,716 | 33,991 |
| GS264 | 35,205,643 | 45,364 | 33,171 |
| GS265 | 34,876,812 | 44,730 | 35,735 |
| GS266 | 34,362,337 | 43,646 | 36,452 |
| GS267 | 34,306,198 | 44,208 | 32,397 |
| GS268 | 34,306,261 | 44,692 | 38,305 |
| GS269 | 32,544,969 | 44,090 | 33,732 |
| GS270 | 35,052,260 | 44,994 | 38,491 |
| GS271 | 29,485,513 | 38,444 | 29,070 |
| GS272 | 32,776,962 | 42,822 | 31,713 |
| GS277 | 35,436,991 | 46,030 | 36,659 |
| GS278 | 32,964,673 | 42,912 | 35,121 |
| GS299 | 36,495,797 | 44,834 | 30,252 |
| GS300 | 35,722,598 | 45,540 | 35,784 |
| GS301 | 34,903,031 | 43,566 | 33,653 |
| GS302 | 35,984,205 | 45,306 | 31,689 |
| GS305 | 36,541,935 | 45,918 | 31,326 |
| GS306 | 38,665,157 | 46,254 | 30,699 |
| GS307 | 37,139,485 | 45,724 | 32,990 |
| GS308 | 36,084,241 | 45,012 | 28,930 |
| GS309 | 37,900,259 | 47,500 | 34,071 |
| GS310 | 21,675,370 | 27,192 | 19,111 |
| GS311 | 32,825,055 | 44,140 | 34,635 |
| GS312 | 22,789,900 | 28,354 | 23,719 |
| GS313 | 32,633,937 | 43,276 | 36,852 |
| GS320 | 36,896,235 | 45,668 | 31,873 |
| GS321 | 35,343,018 | 44,382 | 30,723 |
| GS322 | 35,951,265 | 44,600 | 35,739 |
| GS323 | 35,837,479 | 44,936 | 33,234 |
| GS324 | 35,769,378 | 44,582 | 31,466 |
| GS325 | 35,247,440 | 44,278 | 30,026 |
| GS326 | 35,285,938 | 43,926 | 28,201 |
| GS327 | 36,392,293 | 45,422 | 34,067 |
