# Supplementary material for: Biogeographic and Evolutionary Patterns of Trace Element Utilization in Marine Microbial World
Source: Genomics Proteomics Bioinformatics. 2021 Feb 23;19(6):958–72. doi: 10.1016/j.gpb.2021.02.003 (PMC9402790; doi:10.1016/j.gpb.2021.02.003)
Supplement: Supplementary Table S9 [file mmc18.docx]

**Table S9 List of known metalloprotein (for Cu, Mo, Ni, and Co) and selenoprotein families**

| **Trace element** | **Protein family** |
| --- | --- |
| Cu | Plastocyanin family (plastocyanin, amicyanin, pseudoazurin, halocyanin, *etc*.)  Azurin family (including azurin and auracyanin)  Rusticyanin  Nitrosocyanin  Cytochrome c oxidase subunit I and II  Nitrous oxide reductase  NADH dehydrogenase 2  Cu-Zn superoxide dismutase  Copper amine oxidase  Particulate methane monooxygenase  Nitrite reductase  Multicopper oxidases (CueO, laccase, bilirubin oxidase, phenoxazinone synthase, *etc*.)  Tyrosinase |
| Mo | Sulfite oxidase  Xanthine oxidase  Dimethylsulfoxide reductase  Nitrogenase (Fe-Mo) |
| Ni | Urease  Ni-Fe hydrogenase  Carbon monoxide dehydrogenase  Acetyl-coenzyme A decarbonylase/synthase  Superoxide dismutase SodN  Methyl-coenzyme M reductase  Lactate racemase |
| Co | Methylmalonyl-CoA mutase  Isobutyryl-CoA mutase  Ethylmalonyl-CoA mutase  Glutamate mutase  Methyleneglutarate mutase  D-lysine 5,6-aminomutase  Diol dehydratase  Glycerol dehydratase  Ethanolamine ammonia lyase  B12-dependent ribonucleotide reductase  Methionine synthase  Other methyltransferases: Mta, Mtm, Mtb, Mtt, Mts and Mtv  B12-dependent reductive dehalogenase CprA  LitR/CarH/CarA  PpaA  Epoxyqueuosine reductase |
| Se | Formate dehydrogenase  Selenophosphate synthetase  Coenzyme F420-reducing hydrogenase alpha subunit  Coenzyme F420-reducing hydrogenase delta subunit  Methylviologen-reducing hydrogenase alpha subunit  Glycine reductase selenoprotein A  Glycine reductase selenoprotein B  Peroxiredoxin (Prx)  Thioredoxin (Trx)  Glutaredoxin (Grx)  Heterodisulfide reductase alpha subunit  Thiol:disulfide isomerase-like protein  Thiol:disulfide interchange protein  HesB-like  Proline reductase  Deiodinase-like  Glutathione peroxidase (GPx)  Selenoprotein W-like  Methionine-S-sulfoxide reductase (MsrA)  Fe-S oxidoreductase  DsbA-like  DsrE-like  AhpD-like  Arsenate reductase  Molybdopterin biosynthesis protein MoeB  Glutathione S-transferase  COG0737 UshA  OsmC-like  Rhodanase-related protein  Methylated-DNA-protein-cysteine methyltransferase  UGSC-containing protein  CMD domain containing protein  Arsenite S-adenosylmethyltransferase  Prx-like/Trx-like/Grx-like and Trx-fold proteins  Other hypothetical and predicted selenoproteins |
